# Carcinoma Initiation via Rb Tumor Suppressor Inactivation: A Versatile Approach to Epithelial Subtype-Dependent Cancer Initiation in Diverse Tissues

**DOI:** 10.1371/journal.pone.0080459

**Published:** 2013-12-02

**Authors:** Yurong Song, Debra Gilbert, T. Norene O’Sullivan, Chunyu Yang, Wenqi Pan, Alisan Fathalizadeh, Lucy Lu, Diana C. Haines, Philip L. Martin, Terry Van Dyke

**Affiliations:** 1 Mouse Cancer Genetics Program, Center for Cancer Research, National Cancer Institute, Frederick, Maryland, United States of America; 2 Department of Genetics, University of North Carolina at Chapel Hill, Chapel Hill, North Carolina, United States of America; 3 Department of Cell and Developmental Biology, University of North Carolina at Chapel Hill, Chapel Hill, North Carolina, United States of America; 4 Michigan State University, East Lansing, Michigan, United States of America; 5 Pathology/Histotechnology Laboratory, SAIC-Frederick, Frederick National Laboratory for Cancer Research, Frederick, Maryland, United States of America; 6 Center for Advanced Preclinical Research, SAIC-Frederick, Frederick National Laboratory for Cancer Research, Frederick, Maryland, United States of America; H. Lee Moffitt Cancer Center & Research Institute, United States of America

## Abstract

Carcinomas arise in a complex microenvironment consisting of multiple distinct epithelial lineages surrounded by a variety of stromal cell types. Understanding cancer etiologies requires evaluating the relationship among cell types during disease initiation and through progression. Genetically engineered mouse (GEM) models facilitate the prospective examination of early oncogenic events, which is not possible in humans. Since most solid tumors harbor aberrations in the RB network, we developed an inducible GEM approach for the establishment and assessment of carcinoma initiation in a diverse range of epithelial tissues and subtypes upon inactivation of RB-mediated tumor suppression (RB-TS). The system allows independent assessment of epithelial subtypes that express either cytokeratins (K) 18 or 19. By Cre-dependent expression of a protein that dominantly inactivates RB and functionally redundant proteins p107 and p130, neoplasia could be initiated in either K18 or K19 expressing cells of numerous tissues. By design, because only a single pathway aberration was engineered, carcinomas developed stochastically only after long latency. Hence, this system, which allows for directed cell type-specific carcinoma initiation, facilitates further definition of events that can progress neoplasms to aggressive cancers via engineered, carcinogen-induced and/or spontaneous evolution.

## Introduction

Malignant cancers evolve through mechanisms of selective growth, survival, and invasive properties in a diverse microenvironment. Thus, cancers are heterogeneous with a complex constituency of multiple cell types. Genetically engineered mouse (GEM) models provide a powerful approach to assess both cause/effect relationships and cell type susceptibility. Over the past few decades, progress has been made in identifying molecular aberrations that can contribute to tumorigenesis, thus offering insight into potential causal mechanisms and therapeutic targets. However, since many events have been engineered together and often don’t initiate disease, relatively few studies have explored the etiology of tumor evolution from initiation through progression to advanced disease. Here, we sought to develop “GEM cancer-initiator” models that could be induced to initiate tumorigenesis in a wide variety of epithelial tissues and in distinct subtype compartments for further use in defining tissue and cell-specific mechanisms for carcinoma progression.

Cell subtypes of an epithelium can be characterized by specifically paired cytokeratin (K) expression profiles [1]–[3]. Cytokeratins are cytoskeleton protein intermediate filaments assembled from heterodimeric subunits of acidic type I (K9–K28) and basic type II (K1–K8, and K71–K80) proteins [4]. K18, usually paired with K8, is the first keratin expressed at the eight-cell stage of mouse development [5], [6], followed by K19 and K7 [7]. However, K19, the smallest known acidic keratin, has no known basic type II keratin partner. Although both K18 and K19 are expressed in simple epithelia, they are differentially expressed in subtypes of some epithelia (e.g. K18 in umbrella cells and K19 in basal and intermediate cells of urothelium [2], [3], [8]). Therefore, distinct keratin expression patterns can be utilized as the basis for targeting tumor initiation events to specific epithelial subtypes.

Retinoblastoma 1 (RB) is a negative regulator of proliferation in the eukaryotic cell cycle, particularly during cellular differentiation [9], and the RB pathway plays a critical role in tumorigenesis. Aberrant RB pathway activity, resulting from defects in RB itself, CDKN2A, CCND1, or CDK4, is observed in most solid human cancers [10]. In the few cancer types wherein early human tissue is routinely accessible, such aberrations are often present, suggesting a role in initiation [11]–[15]. However, in some cases, the first association of RB pathway aberration is apparent in more advanced cancer, arguing a role in progression [16]. Indeed, cause and effect relationships likely depend on multiple variables, including the cell and tissue of origin and the stochastic order of causal events, emphasizing the need for model systems to determine plausible roles in disease etiology. Because of functional redundancy among RB and its family members p107 and p130 in most murine cell types [17]–[20], we have used a dominant inactivating protein, T_121_, to inactivate RB tumor suppression (RB-TS) in the mouse [21]–[25]. T_121_ is derived from the N-terminal 121 amino acids of Simian Virus 40 (SV40) large T antigen, which evolved to inactivate the RB-mediated cell cycle brake. When directed by tissue specific promoters in transgenic mice, T_121_ is sufficient to initiate tumorigenesis in prostate [26], mammary [27], ovarian [28] and choroid plexus [29] epithelial cells, as well as in central nervous system astrocytes [30], [31]. The initiation phenotype is dependent on the T_121_ RB/p107/p130 binding site as demonstrated by point mutagenesis [32]. In each case examined thus far, initiation is associated with aberrant proliferation accompanied by apoptosis, and tumor progression has been driven by engineered and/or stochastic aberration of specific cancer-associated molecular networks [28], [31], [33].

Here, we describe transgenic mouse lines in which RB-TS inactivation can be induced in either K19 or K18-expressing epithelial subtypes in a Cre recombinase-dependent fashion. Transgenic lines were established in which Cre-conditional expression of T_121_ was driven under cytokeratin18 or 19 transcriptional control using bacterial artificial chromosome (BAC) transgenes that retain endogenous expression patterns in diverse tissues. Thus, a single strain for each subtype can be used to drive cancer initiation tissue-specifically via organ-specific Cre expression after germline or somatic introduction of a *Cre* transgene. By selective inactivation of RB-TS in K18- or K19-expressing cells, we demonstrate the broad utility of these “cancer-initiator” mice in mechanistic studies of cancer development and show roles for RB-TS inactivation and epithelial subtype specificity in neoplastic initiation within numerous epithelial organs.

## Materials and Methods

### Ethics Statement

This study was carried out in strict accordance with the recommendations in the Guide for the Care and Use of Laboratory Animals of the National Institutes of Health. The protocol was approved by Animal Care and Use Committee (ACUC) of UNC-Chapel Hill (Permit Number: 04-232.0), and National Cancer Institute (NCI)-Frederick (Permit Number: 11-030). All animals in this study were euthanized per the “Guidelines For The Euthanasia of Mouse and Rat Fetuses and Neonates” as defined by the ACUC of UNC-Chapel Hill and NCI-Frederick to minimize pain and suffering.

### Generation of Subtype-specific Keratin-directed BAC Transgenic Mice

**Figure 1:**
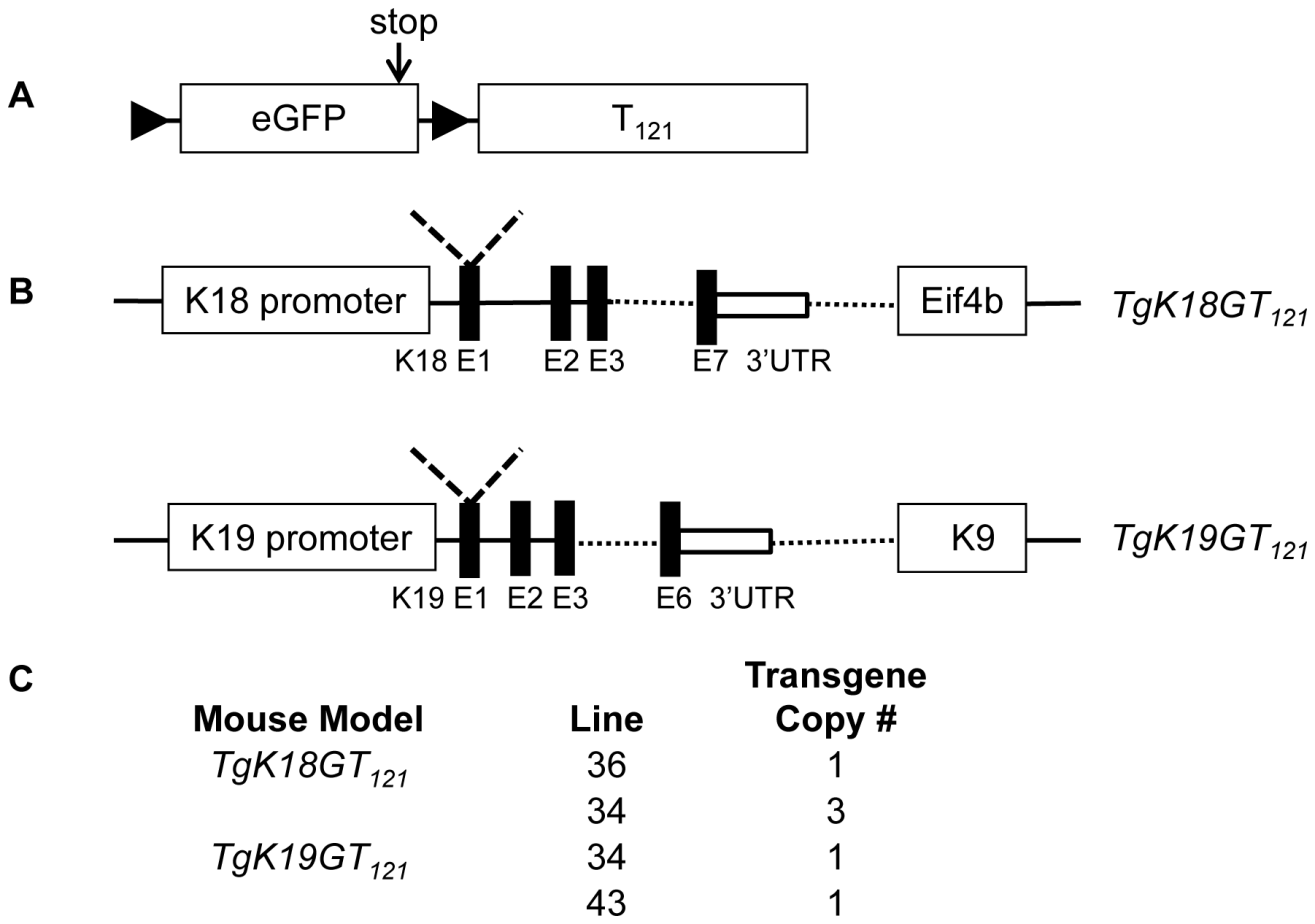
Scheme of transgene constructs and copy number. A. Transgene cassette: An eGFP stop cassette was flanked by loxP sites and the T_121_ gene was placed downstream. B. Keratin bacterial artificial chromosome (BAC) transgene constructs: a transgene cassette (A) was inserted upstream of the start ATG codon in exon 1 of K18 (derived from mouse chromosome (Chr.) 15) and of K19 (derived from mouse Chr 11) in BACs using recombineering. The insertion site for the loxP-eGFP-stop-loxP-T_121_ cassette is indicated by a dashed “V”. Representative genes downstream of keratin genes are indicated in boxes. Solid black bars indicate exons (E). C. Transgene copy number per diploid genome was determined by qPCR.

A floxed eGFP stop T_121_ cassette (transgene cassette, Figure 1A) was derived from the MFT_121_ construct [34]. T_121_ is comprised of the first 121 amino acids (aa) of the SV40 large T antigen followed by 11 missense amino acids resulting from a 31 bp deletion. The transgene cannot express small t antigen due to a second deletion that removes its splice acceptor site [35]. T_121_ inactivates RB and family members p107 and p130 and does not contain the p53 and p300 inactivating domain [36]. The RPCI-22 mouse genomic DNA library was screened using specific probes designed for K18 (Krt1-18 on chromosome 15) and K19 (Krt1-19 on chromosome 11) via the RPCI BAC screening service (Roswell Park Cancer Institute, NY). Positive clones were used for recombineering-directed insertion [37], [38] of floxed eGFP-stop-T_121_ cassettes into exon 1 start codons of K18 and K19. BAC DNA was purified (MTR Scientific, Ijamsville, MD) and injected into fertilized eggs harvested from a B6D2F1 (JAX Laboratory, Bar Harbor, Maine) cross at a concentration of 4 ng/ul without linearization as described [39]. Resulting and subsequent generations of transgenic mice were identified by PCR amplification of a 215-bp fragment using primers 5′ GAATCTTTGCAGCTAATGGACC 3′ and 5′ GCATCCCAGAAGCTCCAAAG 3′ and digit- or ear-derived genomic DNA as template. The cycling profile was: 94°C, 2 minutes; followed by 35 cycles of 94°C, 20 seconds; 62°C, 45 seconds; 72°C, 45 seconds; and a final incubation of 72°C, 2 minutes. Mouse lines were maintained by mating with wild type B6D2F1 mice and therefore are designated as *B6;D2-Tg(K18GT_121_)Tvd* (*TgK18GT_121_*) and *B6;D2-Tg(K19GT_121_)Tvd* (*TgK19GT_121_*).

### Transgenic Breeding Strategies

*TgK18GT_121_* and *TgK19GT_121_* mice were crossed to *β-actin Cre* mice (FVB or C57BL6/NCr background), and *TgK19GT_121_* to *K19CreER* mice (C57BL/6 background, provided by Dr. Guoqiang Gu at Vanderbilt University) [40] to determine the tumor initiation capability upon RB-TS inactivation in K19 and K18 subtypes.

### Transgene Copy Number Determination

Tail genomic DNA was extracted using Qiagen DNeasy Blood and Tissue kit, and quantitative real time PCR was used to detect transgene copy numbers. PCR amplifications were performed in 96-well plates in the ABI 7500 sequence detector. 30 ul samples included 10 ul (50 ng) genomic DNA sample and 20 ul PCR reaction mixture. Amplification was with 10 minutes at 94°C followed by 40 cycles of 15 seconds at 94°C and 1 minute at 60°C. Primers and probe sequences for T_121_ gene were: forward: 5′ CTT TGC AGC TAA TGG ACC TTC 3′, reverse: 5′ TGC CTT TCT CAT CAG AGG AAT 3′, and probe: 5′ Fam TC CCC CAG GCA CTC CTT TCA AGA C Tamra 3′. The endogenous β-actin gene control primers were: forward: 5′ CTG CCT GAC GGC CAG GTC 3′, reverse: 5′ CAA GAA GGA AGG CTG GAA AAG A 3′, and probe: 5′ Fam CA CTA TTG GCA ACG AGC GGT TCC G Tamra 3′. Relative differences of transgene copy numbers were calculated using a standard dCt method with a known amount of control plasmid.

### Histopathology and Immunodetection

Transgenic tissues were dissected at indicated ages and fixed overnight in 10% neutral buffered formalin, transferred to 70% ethanol, routinely processed, and embedded in paraffin. Tissues were sectioned for 10 successive layers at 5 µm intervals except lymphoid tissues (4 µm) and stained with haematoxylin and eosin (H&E) for histopathological examination. Immunohistochemistry (IHC) and immunofluorescence (IF) analyses were performed on formalin-fixed paraffin-embedded sections as previously described [26]. Antibodies included: anti-K8/18 (1∶500, guinea pig polyclonal, GP11, Progen Biotechnik, GMBH, Heidelberg, Germany), anti-K19 (1∶200, rabbit monoclonal, Epitomics, CA), anti-K14 (1∶1000, rabbit polyclonal, pRB-155P, Covance), anti-GFP (1∶500, b-2, mouse monoclonal, Santa Cruz), anti-SV40 T antigen (1∶100, mouse monoclonal, DP02-200UG, Calbiochem), and anti-Ki-67 (1∶500, rabbit polyclonal, 06-570, BD Pharmingen, San Diego, CA). For double IF staining, the first primary antibody (anti-eGFP or anti-SV40 T antigen) was incubated overnight followed by the second primary antibody (anti-K19, anti-K14, or anti-K18) incubation for 2 hours at room temperature. Mixed Alexa fluor 488 and 594 (1∶200 dilution, Life Technologies) served as secondary antibodies. Images were captured using light, immunofluorescence, or Zeiss confocal microscopes. Apoptosis levels were detected in sections using the terminal deoxynucleotidyltransferase-mediated dUTP-biotin nick end labeling (TUNEL) assay (ApopTag Peroxidase *In Situ* Apoptosis Detection Kit, S7100 Millipore) following the manufacturer’s instruction [26]. To quantify Ki-67 positive cells, random 5–10 Ki-67-positive fields with the same magnification were acquired using a Zeiss microscope, and Ki-67 positive and negative cells were counted manually. Positivity was calculated as the average percentage of total cells.

### Statistical Analyses

Student t or Mann-Whitney rank sum tests were performed to evaluate the statistical significance among genotypes and tissues. P<0.05 was considered statistically significant.

## Results

### Transgene Expression Accurately Reflects Epithelial Subtype Specificity

To target RB-TS inactivation to epithelial subtypes, we employed keratin regulation within BACs to drive conditional transgene expression. A Cre-conditional loxP-eGFP-stop-loxP (LSL) T_121_ cassette (Figure 1A) was introduced into the first exon of either the K18 or the K19 gene within respective BACs to generate *TgK18GT_121_* and *TgK19GT_121_* transgenic mice (Figure 1B). Thus, eGFP was expressed under K18 or K19 regulation until expression/activation of Cre, which mediated *eGFP* deletion and induced T_121_ expression. Two transmitting founder lines for each construct were generated and characterized. As assessed by quantitative real time PCR, mice harbored one transgene copy in lines *TgK19GT_121_-34* and *-43,* and *TgK18GT_121_-36*, and 3 transgene copies in line *TgK18GT_121_-34* (Figure 1C).

To detect endogenous K19 and K18 expression, and thus the patterns for accurate transgene regulation, wild type epithelial tissues were examined by IHC or IF. Consistent with reported results [2], [3], [8], both K19 and K18 were widely expressed in a variety of mouse epithelial tissues (Figure S1 and Table S1). *TgK18GT_121_* and *TgK19GT_121_* eGFP transgene expression was visualized in whole tissues using a stereo fluorescence microscope (SteREO Discovery.V20, Carl Zeiss Meditec, Inc). Strong green fluorescence was observed in some tissues of both lines of *TgK19GT_121_* mice (e.g. intestine, stomach, urinary bladder, and gallbladder), but weak or no green fluorescence in others (e.g. mammary gland, ovary, and pancreas, Figure S2). Many tissues showed strong green fluorescence in both lines of *TgK18GT_121_* mice (e.g. intestine, stomach, thymus, mammary gland, ovary, salivary gland, urinary bladder and vas deferens, epididymis, and gallbladder), but not in pancreas, lung, and liver (Figure S3). In *TgK19GT_121_* mice, immunostaining of tissue sections consistently showed strong and widespread eGFP expression in intestine, stomach, urinary bladder, gallbladder, and renal pelvis epithelium. Readily detectable staining was limited to a smaller percentage of cells in lung, mammary gland, and prostate (Figure S4 and Table S1). Other tissues had very weak or no detectable eGFP expression (Table S1). In *TgK18GT_121_* mice, eGFP expression was widespread in most K18-positive epithelial tissues (Figure S5 and Table S1). Importantly, based on double IF, eGFP was co-expressed with respective endogenous keratins in *TgK19GT_121_* (Figure S4) and *TgK18GT_121_* mice (Figure S5), but excluded from K14 cells (Figure S6). However, not all K19 or K18 positive cells expressed eGFP. Both lines of *TgK19GT_121_* mice showed similar eGFP expression. Tissues from *TgK18GT_121_-34* mice showed more intense eGFP staining compared to those from *TgK18GT_121_-36* mice (data not shown), possibly due to the higher transgene copy number in line 34 (Figure 1C).

### RB-TS Inactivation is Induced in Specific Epithelial Subtypes

**Figure 2:**
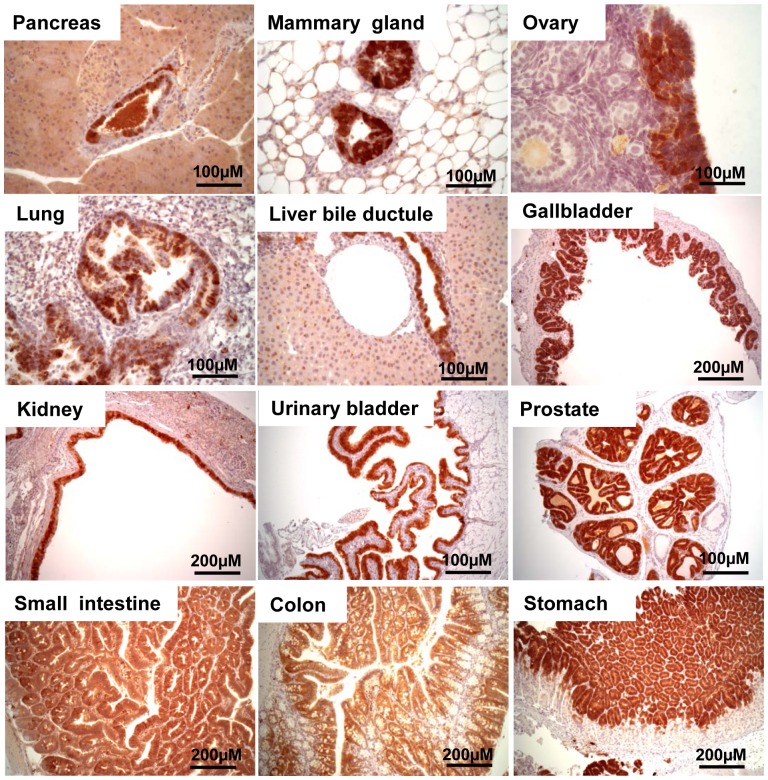
T_121_ expression is induced in tissues of 2 month-old *TgK19GT_121_; β-actin Cre* mice by IHC. Strong brown staining indicates positive T_121_ expression.

To determine whether T_121_ expression could be induced in a broad range of epithelial tissues with subtype specificity and to evaluate the effect of RB-TS inactivation, we crossed hemizygous transgenic mice from all lines to transgenic *β-actin Cre* homozygous mice. Likely due to broad RB-TS inactivation in K19 expressing embryonic cells (see below), some embryonic lethality was observed in *TgK19GT_121_; β-actin Cre* mice. Live-born bi-transgenic mice were under-represented relative to littermate controls (34% vs. 66% for *TgK19GT_121_-34*, 45% vs. 55% for *TgK19GT_121_-43*, respectively; Table S2), suggesting that RB function in K19 cells may be critical for development. In the surviving mice, consistent with the breadth of endogenous K19 expression (Figure S1), T_121_ expression was readily observed in a variety of tissues first assessed at 2 months of age (Figure 2), even though eGFP expression had been low or below the IHC detection level in some of these tissues (e.g. ovary, mammary gland and pancreas, Figures S2 and S4, and Table S1). Importantly, as eGFP was faithfully expressed in K19 cells, T_121_ was also expressed in K19-positive cells (not shown), but excluded from K14 cells (Figure S6). T_121_ expression levels were comparable between *TgK19GT_121_-34; β-actin Cre* and *TgK19GT_121_-43; β-actin Cre* mice (not shown).

**Figure 3:**
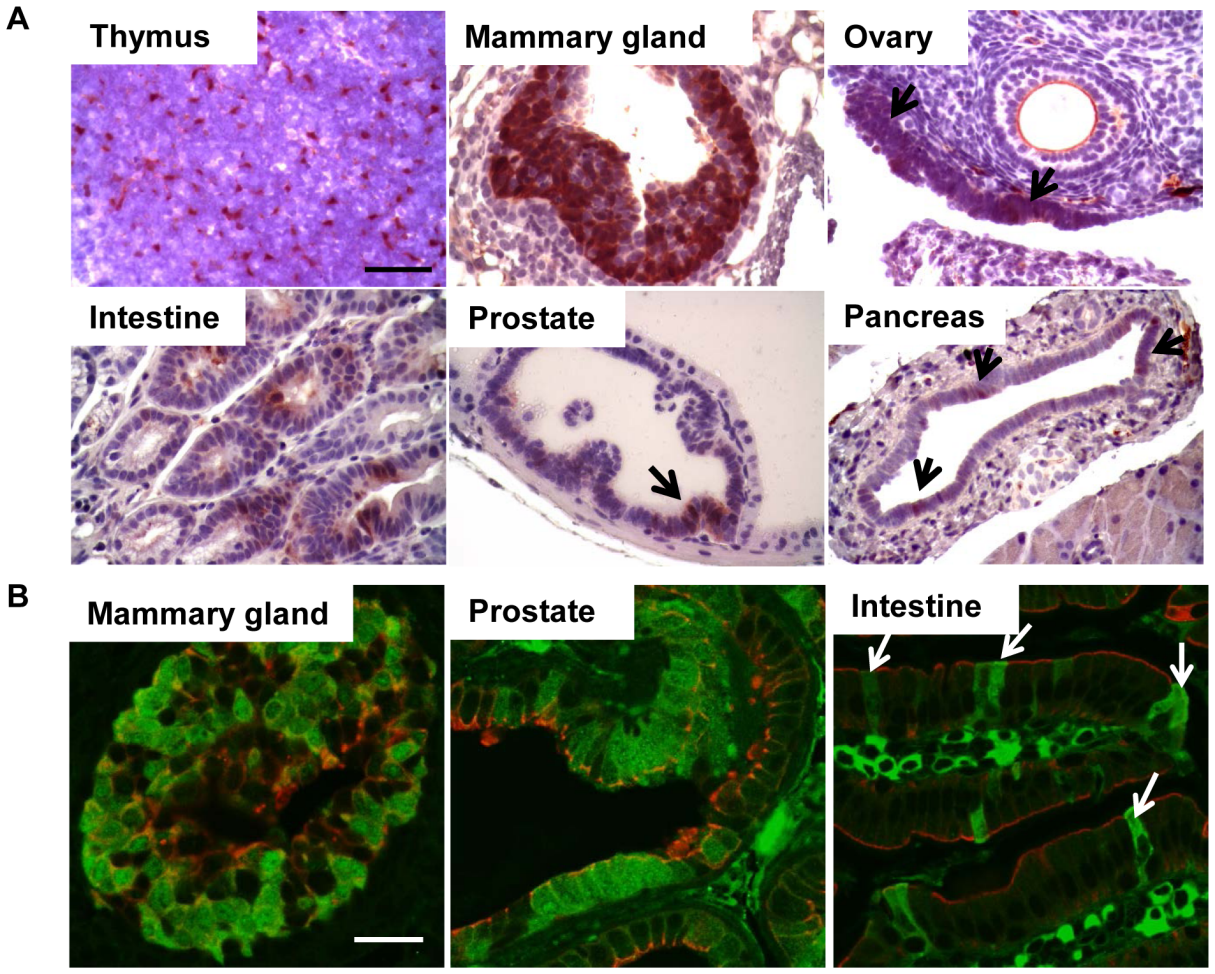
T_121_ expression is induced in tissues of 2-month old *TgK18GT_121_; β-actin Cre* mice. A. T_121_ expression by IHC. Black arrows indicate T_121_ positive cells. Scale bar = 50 µM. B. Double immunofluorescence staining of T_121_ as green and endogenous K18 as red in tissues shown as merged confocal images. White arrows indicate intestinal epithelial cells positive for both endogenous K18 and T_121_. Scale bar = 25 µM.

Contrary to *TgK19GT_121_; β-actin Cre* mice, both lines of *TgK18GT_121_; β-actin Cre* mice developed with normal Mendelian frequencies (not shown). T_121_ was widely expressed in K18 expressing epithelium of some tissues of 2 month old *TgK18GT_121_; β-actin Cre* mice (e.g. thymus, mammary gland, and ovary), but showed limited induction in others, albeit in the appropriate cells (e.g. intestine, prostate, and pancreas; Figure 3), even though eGFP expression had been more widespread in some cases (Figures S3 and S5, and Table S1). Importantly, T_121_ was expressed in K18 positive cells (Figure 3B), but excluded from K14 cells (Figure S6). The intensity of T_121_ expression was comparable between *TgK18GT_121_-34; β-actin Cre* and *TgK18GT_121_-36; β-actin Cre* lines (not shown) although eGFP expression was higher in *TgK18GT_121_-34* than *TgK18GT_121_-36* mice (not shown). This observation may reflect somatic Cre-mediated copy number reduction from a tandem gene array.

### RB-TS Inactivation Causes Neoplasia

**Figure 4:**
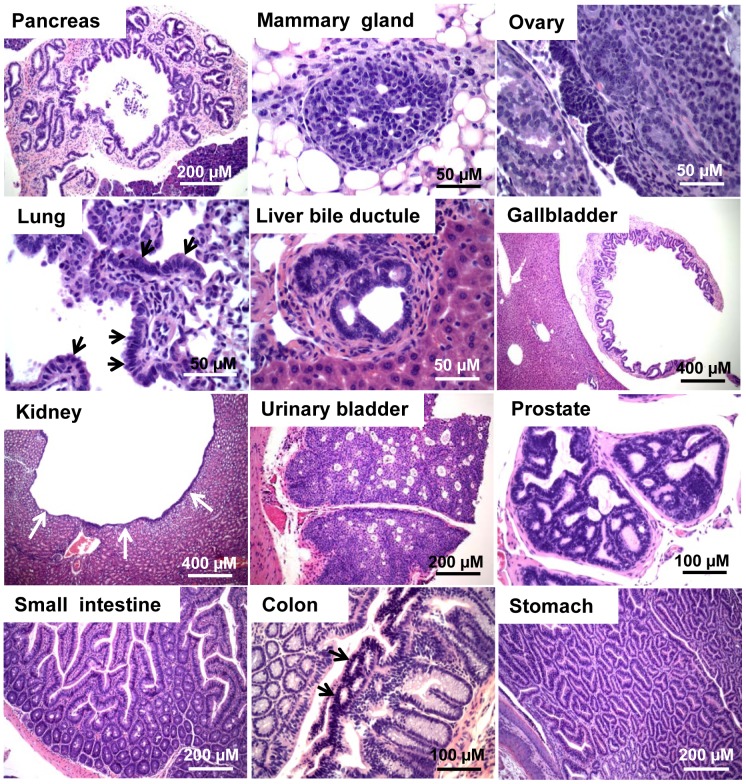
Neoplastic lesions in *TgK19GT_121_;* *β-actin Cre* mice. Hyperplasia was observed in most tissues (lung and colon: indicated by black arrows). Multifocal ductal hyperplasia was observed in pancreas, as well as mouse prostate intraepithelial neoplasia (mPIN) in prostate, and hydronephrosis as indicated by white arrows in kidney. All samples were stained with H&E.

**Figure 5:**
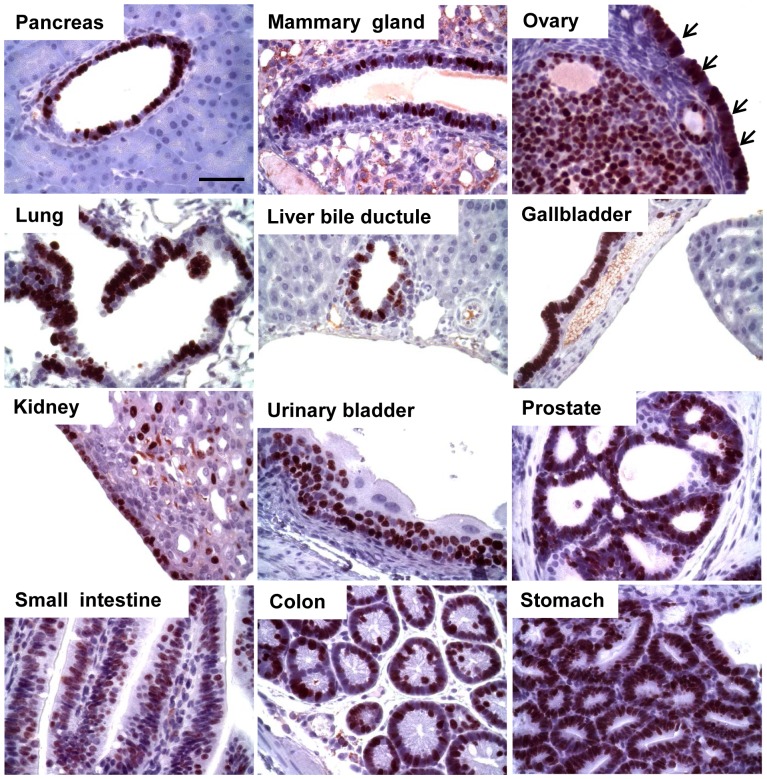
Proliferation in T_121_-expressing *TgK19GT_121_;* *β-actin Cre* tissues. Proliferation was assessed by Ki-67 IHC (brown) in tissues of 2 months old mice. Arrows indicate ovarian surface epithelial cells (OSECs) positive for Ki-67. Scale bar = 50 µM.

**Figure 6:**
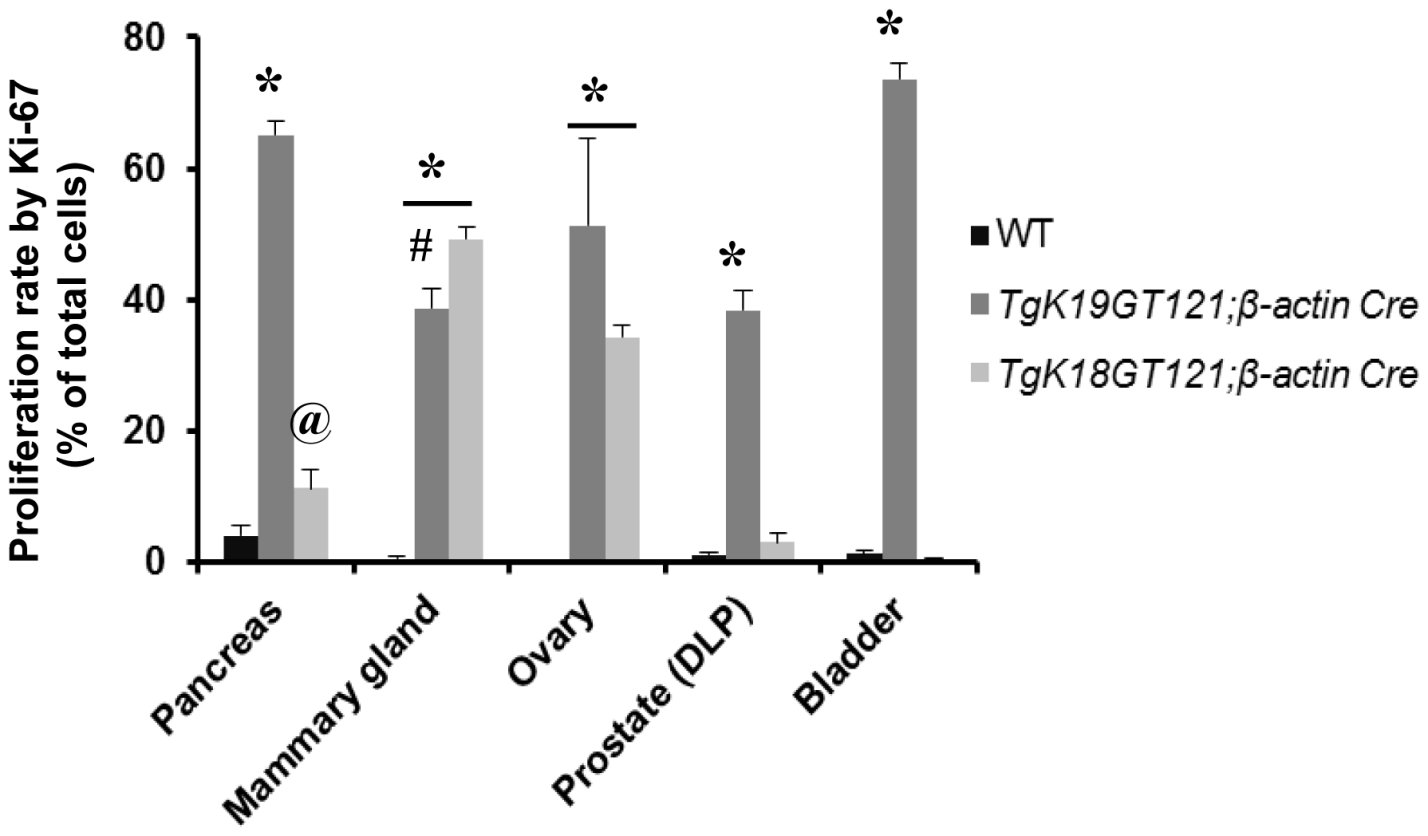
Proliferation rates in tissues of *TgK19GT_121_;* ** *β-actin Cre* and *TgK18GT_121_;* ** *β-actin Cre* mice. Proliferation rates of pancreas, mammary gland, ovary, prostate (dorsolateral lobe), and bladder were quantified on tissue sections stained with Ki-67. *Significantly different from WT control (p<0.001); #significantly different between *TgK19GT_121_; β-actin Cre and TgK18GT_121_; β-actin Cre* (p = 0.007); @ significantly different between *TgK18GT_121_; β-actin Cre* and WT (p = 0.043).

Compared to wildtype mice (Figure S7A), all *TgK19GT_121_-43; β-actin Cre* mice developed multifocal hyperplastic lesions in most K19 positive epithelial tissues [e.g. pancreas (pancreatic ductal epithelium), mammary gland, ovary (ovarian surface epithelial cells, OSECs), lung bronchiole, liver bile ductule, gallbladder, kidney (renal pelvis epithelial cells), urinary bladder, prostate, small intestine, colon, stomach glandular foveolar cells; Figure 4 and Table 1]. This phenotype was concurrent with high proliferation rates in the affected cells (Figures 5 and 6) compared to wildtype tissues (Figures 6 and S8). As in previous reports of limited tissues [26], [27], [30], increased proliferation in tissues of *TgK19GT_121_; β-actin Cre* mice was accompanied by a significant elevation of apoptosis (Figure S9) compared to that of wildtype mice (Figure S10). These results indicate that RB is important for suppressing hyperplasia of K19 positive cells. Some *TgK19GT_121_; β-actin Cre* mice developed life-threatening bilateral or unilateral hydronephrosis starting as early as 2 months of age (Figure S11A). The hydronephrosis was interpreted to be secondary to marked hyperplasia of transitional cell epithelium at the junction of the renal pelvis and ureter resulting in obstruction of urine outflow from the kidney (Figure S11A). Other mice from line 34 had to be euthanized due to enlarged thymuses, which led to life- threatening thoracic pressure, at 7 months of age (Figure S11B).

**Table 1:** Summary of histopathological findings in *TgK19GT_121_; β-actin Cre* and *TgK18GT_121_; β-actin Cre* mice.

Lesions	*TgK19GT_121_; β-actin Cre* * (n = 15, 6 m and 9 f)	*TgK18GT_121_; β-actin Cre* * (n = 10, 2 m and 8 f)
Lung bronchiole hyperplasia	15 (100%)	0
Intestine hyperplasia†	15 (100%)	4 (40%)
Stomach, glandular foveolar cells hyperplasia	15 (100%)	0
Gallbladder epithelium hyperplasia	15 (100%)	0
Prostate hyperplasia and mPIN†	6 (100%)	2 (100%)
Oviduct hyperplasia	9 (100%)	N.D.
Kidney hydronephrosis	11(73%)	0
Liver bile ductule hyperplasia	10 (67%)	0
Ureter hyperplasia	9 (60%)	N.D.
Urinary bladder hyperplasia†	8 (53%)	1 (10%)
Mammary epithelium hyperplasia†	4 (44%)	8 (100%)
Pancreatic ductal epithelium hyperplasia#	4 (27%)	0
Adrenal hyperplasia	3 (20%)	3 (30%)
Thymus hyperplasia†	2 (13%)	10 (100%)
Uterus hyperplasia	1 (7%)	2 (20%)
Ovarian surface epithelium hyperplasia	4 (44%)	4 (50%)

* Average age of *TgK19GT_121_; β-actin Cre* mice analyzed here was 123 days (2–6 months, mainly line 43) and *TgK18GT_121_; β-actin Cre* mice 130 days (1–6 months, both lines). Three *TgK19GT_121_; β-actin Cre* mice were euthanized due to kidney hydronephrosis at 2 months of age. All *TgK18GT_121_; β-actin Cre* mice were euthanized due to thymic masses at 1–6 months of age.

† The onset and extent of lesions were different between *TgK19GT_121_; β-actin Cre* and *TgK18GT_121_; β-actin Cre* mice (see text for details).

# multifocal hyperplasia. m: male; f: female; N.D.: not determined.

**Figure 7:**
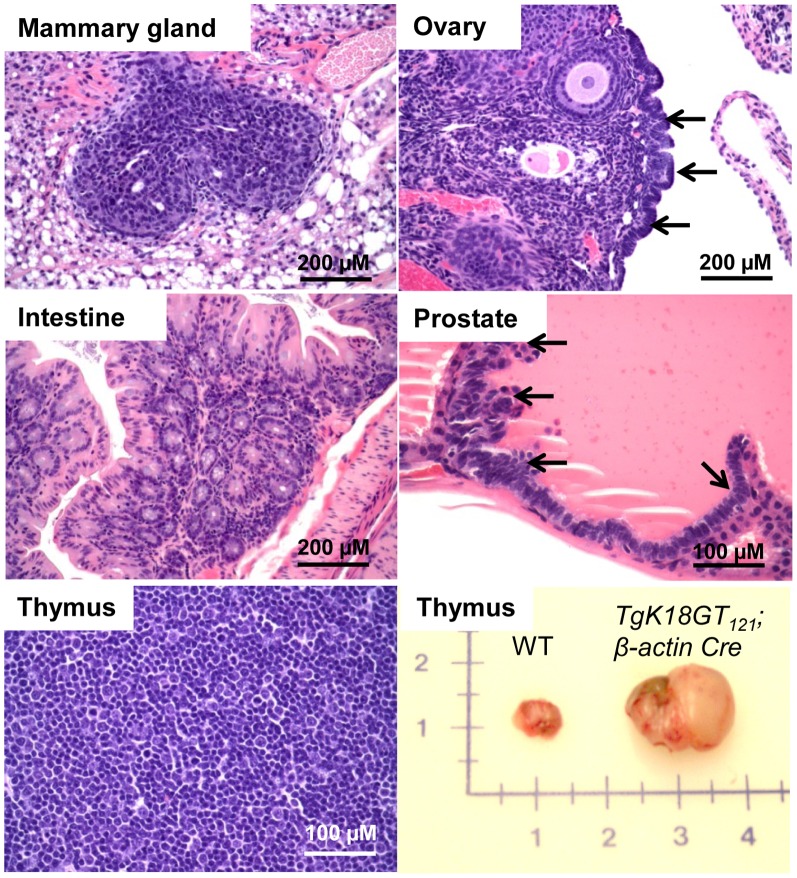
Neoplastic lesions in *TgK18GT_121_; β-actin Cre* mice. Hyperplasia was observed in some tissues (ovary and prostate: indicated by arrows). All mice were euthanized at 1–7 months of age due to life-threatening thoracic pressure caused by enlarged thymuses. All samples were stained with H&E.

**Figure 8:**
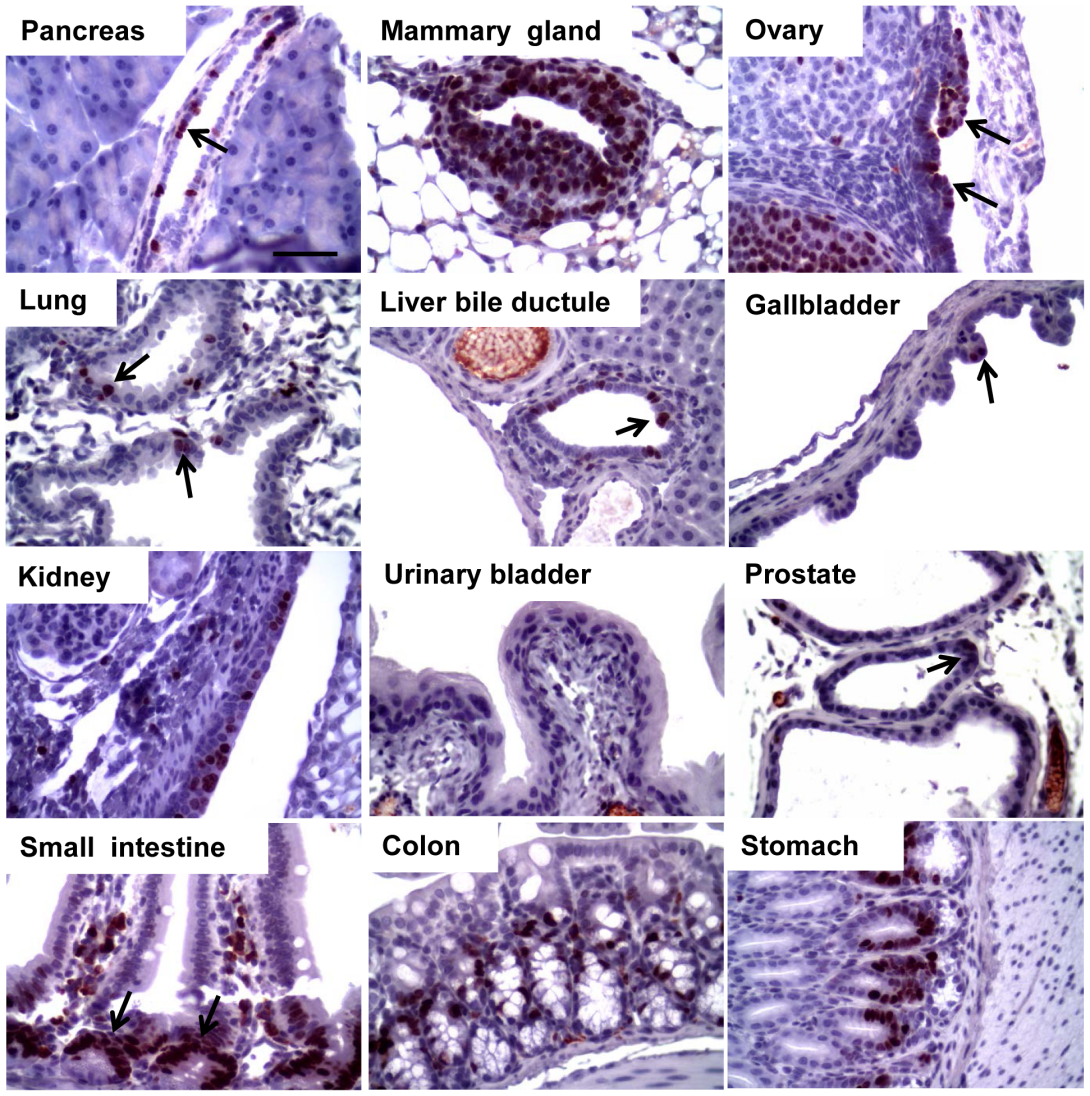
Proliferation in T_121_-expressing *TgK18GT_121_;* ** *β-actin Cre* tissues. Proliferation was assessed as in Figure 5 in 2 month old mice. Similar proliferation levels were observed in small intestine, colon, stomach and ovarian follicle as that of wildtype mice shown in Figure S8. Black arrows indicate Ki-67 positive cells. Scale bar = 50 µM.

Similar to *TgK19GT_121_; β-actin Cre* mice, inactivation of RB-TS in K18-positive cells predominantly led to hyperplasia (Figure 7 and Table 1), which was correlated with T_121_ expression in these tissues (Figure 3). With broad induction by the *β-actin Cre* allele, both *TgK18GT_121_-34* and *TgK18GT_121_-36* mice developed severe thymic epithelial hyperplasia and subsequent lymphoid hyperplasia (Table 1 and Figure 7). Immunostaining indicated high T_121_ expression in K18 cortical thymic epithelium (Figure S12B) associated with high proliferation (based on Ki-67 IHC) and apoptosis (TUNEL) rates (Figure S12C). Because these mice were compromised due to life-threatening thoracic pressure from enlarged thymuses (Figure S12A) and required euthanasia by 6 months of age, *TgK18GT_121_; β-actin Cre* phenotypes in other tissues could not be examined past this age. At 1–6 months of age, mild focal hyperplasia was observed in mammary gland, ovarian surface epithelium, intestine, and prostate (Figure 7 and Table 1), which correlated with elevated proliferation rates and increased apoptosis (Figures 6, 8, and S13). For the most part, when compared to *TgK19GT_121_; β-actin Cre* mice, the extent of hyperplasia was less widespread in *TgK18GT_121_; β-actin Cre* mice, which correlated with the limited extent of T_121_ expression in these tissues. High proliferation rates were observed in both K19 and K18 positive mammary epithelia and ovarian surface epithelia (38.6% vs. 49.2% in mammary, and 51.2% vs. 34.2% in ovary, respectively, Figure 6), which could be explained by co-expression of K19 and K18 in these tissues (Figure S1). However, this is not the case for other sites of co-expression (e.g. prostate, Figure 6), suggesting that tissue-specificity may play a role in Cre recombination efficiencies.

### Inactivation of RB-TS in Adult-restricted K19+ Cells is Sufficient for Tumor Initiation

To circumvent the developmental defects observed in *TgK19GT_121_; β-actin Cre* mice and to demonstrate the utility of these models for studying cancer development in adult organs, we crossed *TgK19GT_121_-34* to tamoxifen-inducible *K19CreER* mice [40]. In contrast to *TgK19GT_121_; β-actin Cre* mice, *TgK19GT_121_;K19CreER* mice were born with expected Mendelian frequencies (not shown), further supporting the critical role of RB-TS in K19 positive cells during mouse development. Mice were induced with tamoxifen at 6–8 weeks of age, and T_121_ expression was assessed by IHC. T_121_ was first readily detectable in gallbladder at 2 weeks post induction (not shown) and was widespread in various tissues by 4–6 weeks post induction (Figure S14A). Moreover, T_121_ was co-expressed with K19 (Figure S14B). Consistent with expression of eGFP prior to Cre expression, and, typical of transgene expression, T_121_ was expressed in a subset of K19 positive cells within a given tissue.

**Figure 9:**
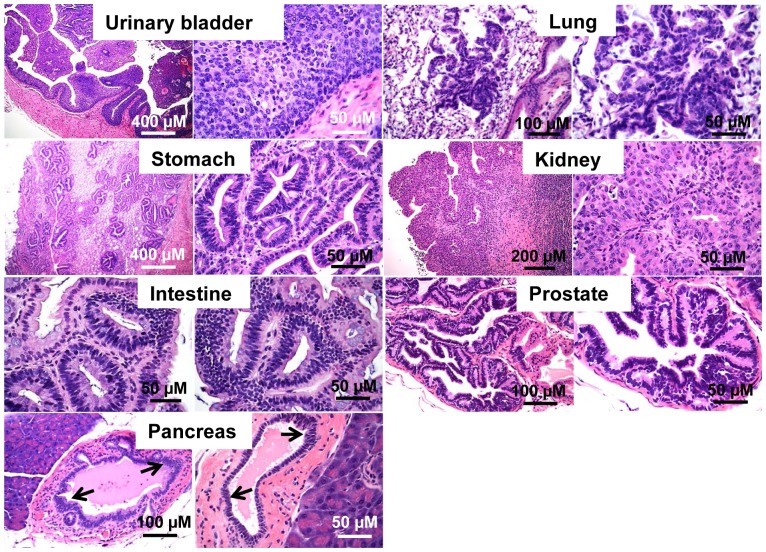
Neoplastic lesions in tamoxifen-induced *TgK19GT_121_; K19CreER* tissues. Urinary bladder adenoma, lung adenoma, stomach adenocarcinoma, renal carcinoma, and gut intraepithelial neoplasia (GIN), and mPIN lesions in prostate are shown at low and high magnifications. Mild pancreatic duct hyperplasia was observed in pancreas as indicated by arrows. All samples were stained with H&E.

To determine whether RB-TS inactivation predisposed adult K19-expressing epithelial cells to tumorigenesis, tamoxifen-induced *TgK19GT_121_;K19CreER* mice were aged. Consistent with the general hyperplastic phenotype observed in *TgK19GT_121_; β-actin Cre* mice, a high frequency of atypical hyperplasia was observed in most epithelial tissues (e.g. stomach glandular epithelium 85.7% in males and 90% in females, and lung bronchiole epithelium 92.9% in males and 90% in females; Table 2), consistent with increased proliferation rates (not shown). While a low frequency of malignant neoplasia did occur in some tissues (e.g. stomach adenocarcinoma 13.3% in males and 14.3% in females; Table 3 and Figure 9), low grade lesions present in most organs indicated that RB-TS inactivation was sufficient to initiate but not to progress disease. This is consistent with our previous results in mammary [27], prostate [26], choroid plexus [29] and ovarian epithelium [28], and brain astrocytes [30], [31]. Similar to *TgK19GT_121_; β-actin Cre* mice, renal hydronephrosis was also observed in 75% of males and 40% of females of induced *TgK19GT_121_;K19CreER* mice at 9–16 months post induction (Table 2). However, unlike *TgK19GT_121_; β-actin Cre* mice, the cause of death was bladder obstruction due to high expression of K19 in intermediate and basal cells of bladder epithelium (Figure S7B) and a high frequency of adenoma development in the bladder (Table 3 and Figure 9). Interestingly, we observed earlier onset of urinary bladder adenoma in males compared to females (9 months vs. 14 months post induction; Table 3). The underlying mechanism is unknown.

**Table 2:** Summary of low grade lesions in tamoxifen-induced *TgK19GT_121_-34;K19CreER* mice.

	Male (>9 mo PI)		Female (>8 mo PI)	
Lesions	n (lesion/total)	Frequency (%)	n (lesion/total)	Frequency (%)
Lung bronchiole hyperplasia	13/14	92.9	9/10	90.0
Colon GIN	11/12	91.7	10/10	100.0
Stomach (glandular) atypical hyperplasia	12/14	85.7	9/10	90.0
Kidney hydronephrosis	12/16	75.0	4/10	40.0
Prostate mPIN	10/16	62.5		
Duodenum GIN	3/5	60.0	0/3	0
Gallbladder atypical hyperplasia	8/14	57.1	3/10	30.0
Cecum GIN	4/13	30.8	1/10	10.0
Urinary bladder atypical hyperplasia*	0/17	0	3/11	27.3

Histopathology was analyzed in males (9–14 months post induction (PI)) and females (8–16 months PI). GIN: gut intraepithelial neoplasia; mPIN: mouse prostate intraepithelial neoplasia. mo: month.

* All males and 8 females developed high grade lesions (adenoma), which are summarized in Table 3.

**Table 3:** Summary of neoplastic lesions developed in tamoxifen-induced *TgK19GT_121_-34;K19CreER* mice.

	Male (>9 mo PI, n = 15)		Female (>14 mo PI, n = 7)	
Lesions	n	Frequency (%)	n	Frequency (%)
Urinary bladder adenoma	15	100.0	5	71.4
Stomach (glandular) adenocarcinoma	2	13.3	1	14.3
Lung adenoma (alveolar/bronchiolar)	2	13.3	2	28.6
Renal papilloma/carcinoma	2	13.3	0	0
Ureter/urethra carcinoma	4	26.7	1* (8 mo PI)	14.3
Ureter adenoma	1	6.7	0	0

Males developed neoplastic lesions at 9 months post induction (PI), while females at 14 months PI except one at 8 months PI (*labeled). mo: month.

## Discussion

Studies utilizing tissue-specific gene targeting in genetically engineered mouse models have contributed extensively to our understanding of cancer complexity and the molecular and cellular basis of tumorigenesis. Here we describe the generation of transgenic mouse models (*TgK18GT_121_* and *TgK19GT_121_*) using keratin gene transcriptional regulators to drive conditional epithelial subtype-specific inactivation of RB-TS. High fidelity of respective keratin-specific expression patterns was achieved using BAC transgenes that were also engineered for assessing tissues of interest via reporter GFP expression. We demonstrate that these models can be used in combination with specific Cre drivers to initiate tumorigenesis in a wide range of epithelial tissues in either K19 or K18 expressing subtypes. We further demonstrate that RB-TS inactivation can indeed elicit pre-cancerous lesions cell autonomously in all tested epithelial tissues, including upon induction strictly in adult organs, and that early neoplasia is uniformly associated with aberrant proliferation and apoptosis.

Using subtype-specific targeting, a broad range of K18 or K19 positive subtypes within epithelial tissues were susceptible to neoplastic induction by RB-TS inactivation. Neither *TgK18GT_121_; β-actin Cre* mice (up to 6 months) nor *TgK19GT_121_; β-actin Cre* mice (up to 8 months) developed adenomas or carcinomas. These results confirm that RB-TS inactivation in affected epithelia only initiates a neoplastic phenotype. Based on previous studies of tumor progression in T_121_-initiated cells, we expect that additional lesions in specific cancer genes will be required to drive this early neoplastic phenotype into respective cancers. Indeed, we have demonstrated this case using the K18-driven system in ovarian surface epithelium, wherein lesions disrupting p53 function facilitate progression to metastatic stage IV serous ovarian cancer [28], and in mammary epithelium, where *Trp53* and *BRCA1* deletion progresses hyperplasia to adenocarcinoma (Van Dyke et al., unpublished). Using the K19-driven system, we have also observed that mammary hyperplasia can progress with additional *PTEN* deletion (Song and Van Dyke et al., unpublished).

In comparing results from K18 and K19-driven models, most tissues showed a potential subtype-dependency with respect to the extent of hyperplasia/dysplasia. This observation was independent of transgene insertion site, since the same results were obtained regardless of the transgenic line within each genotype. However, the interpretation that K18 and K19 expressing epithelial subtypes are differentially responsive to RB-TS inactivation is confounded by the differential transgene induction rate by *β-actin Cre* in K18 and K19-driven models. Indeed, the breadth of oncogene expression, and possibly cellular levels, was directly correlated with the extent of neoplasia induced. *TgK19GT_121_*; *β-actin Cre* epithelial tissues showed wide spread T_121_ induction and neoplasia, while T_121_ was induced only in a subset of cells in most *TgK18GT_121_*; *β-actin Cre* tissues and resulted in minimal neoplasia. This outcome may be due to tissue-dependent Cre recombination efficiencies and/or to temporal differences in K18 and K19 expression onset, since not every *TgK18GT_121_*; *β-actin Cre* tissue showed low T_121_ induction. Whatever the explanation, this study could not distinguish susceptibility between K19 and K18 subtypes in most tissues. Mammary epithelium and ovarian surface epithelium are exceptions to this outcome in that RB-TS inactivation under both K18 and K19 regulation led to overt hyperplasia due to co-expression of K18 and K19 in these tissues, an outcome reflecting the expected endogenous keratin expression patterns. Of note, the K18-driven model, in which limited expression within specific cell populations can be induced, will be valuable for cancer initiation studies, since this situation mimics the limited stochastic initiation that is thought to occur in human cancers. Furthermore, focal induction via viral Cre injection in specific tissue might be the best approach to assess the adult induction of RB-TS inactivation in K18 cells in tissues other than thymic epithelium. Overall, these “GEM cancer-initiator” models provide useful tools with which to further define tissue and cell-specific mechanisms for carcinoma progression via engineered, carcinogen-induced and/or spontaneous evolution.

## Supporting Information

Figure S1 **Endogenous K19 and K18 expression in wildtype mice.** A. Endogenous K19 (brown, Scale bar = 50 µM) and K18 (red, Scale bar = 25 µM) expression was detected by immunohistochemistry (IHC) and immunofluorescence (IF), respectively. B. Endogenous K19 expression (brown) was detected by IHC. Scale bar = 50 µM.
(TIF)

Figure S2 **GFP expression in** ***TgK19GT_121_*** **tissues.** GFP fluorescence was visualized using SteREO Discovery.V20 Zeiss microscope. Indicated regions with grossly detectable expression include colon, cecum, small intestine (purple arrow), stomach (*), urinary bladder (blue arrow), vas deferens (white arrows), seminal vesicle (red arrow), and gallbladder (§). Expression was not observed by this method in prostate (#), liver (&), mammary gland, ovary (©), and pancreas (¤). Scale bar = 2000 µM.
(TIF)

Figure S3 **GFP expression in** ***TgK18GT_121_*** **tissues.** GFP fluorescence was observed in intestine (small intestine, cecum, and colon), stomach (*), thymus, mammary gland, ovary, salivary gland, urinary bladder (blue arrows), vas deferens (white arrows), epididymis (yellow arrow), gallbladder (§), but not in prostate (#), testis (gray arrow), pancreas, lung, and liver (&) using SteREO Discovery.V20 Zeiss microscope.
(TIF)

Figure S4 **Co-expression of transgenic GFP and endogenous K19 in** ***TgK19GT_121_*** **tissues.** GFP (green) and endogenous K19 (red) were co-stained by IF and shown as confocal merged images. Umbrella cells (*) in urinary bladder epithelium were negative for K19 and GFP. Scale bar = 25 µM.
(TIF)

Figure S5 **Co-expression of transgenic GFP and endogenous K18 in** ***TgK18GT_121_*** **tissues.** GFP (green) and endogenous K18 (red) were co-stained by IF and shown as confocal merged images. White arrows indicate ovarian surface epithelial cells (OSECs), which co-expressed K18 and eGFP. Red arrows indicate OSECs that are negative for transgenic eGFP, but positive for endogenous K18, indicating mosaic expression typical of transgenes and observed to varying extents in all tissues. Scale bar = 25 µM.
(TIF)

Figure S6 **Exclusion of transgene expression from K14 cells in mammary glands.** K14 was double immunostained with eGFP in *TgK18GT_121_* and *TgK19GT_121_* or T_121_ in *TgK18GT_121_; β-actin Cre* and *TgK19GT_121_; β-actin Cre* mammary glands. Dapi was used to counterstain nuclei. *indicates the high background staining of eGFP in mammary glands of *TgK19GT_121_* mice.
(TIF)

Figure S7 **Wildtype mouse tissue architecture.** A. Histology of wildtype tissues by H&E staining. B. Double immunostaining of K19 and K18 or K19 and p63 in urinary bladder. White arrows indicate umbrella cells negative for both K19 and p63. Scale bar = 25 µM.
(TIF)

Figure S8 **Proliferation in wildtype tissues.** Proliferation was assessed by Ki-67 IHC (brown) and was either low or undetectable in most tissues. Normal small intestine, colon, stomach, and ovarian follicle proliferation was readily detected. Scale bar = 50 µM.
(TIF)

Figure S9 **Apoptosis in T_121_-expressing** ***TgK19GT_121_; β-actin Cre*** **tissues.** Apoptosis (brown) was assessed by TUNEL in tissues of 2 months old mice. Methyl green was used to counterstain nuclei. Scale bar = 50 µM.
(TIF)

Figure S10 **Apoptosis in wildtype tissues.** Apoptosis, evaluated as in Figure S9, was minimal in tissues of 2 months old wildtype mice. Scale bar = 50 µM.
(TIF)

Figure S11 **Renal and thymic phenotypes in** ***TgK19GT_121_;*** ** ***β-actin Cre*** **mice.** A. Left: Gross morphology of unilateral hydronephrosis shows relative normal size of left kidney and cystic right kidney; Middle: Histopathology of the renal pelvis/ureter junction revealed by H&E staining; Right: proliferation of the renal pelvis/ureter junction by Ki-67 IHC. Marked hyperplasia of transitional cell epithelium in the junction resulted in obstruction of urine outflow from the kidney. Most mice were euthanized due to hydronephrosis at 2–7 months. B. Some *TgK19GT_121_-34; β-actin Cre* mice also developed life-threatening thymic masses (left) at 7 months of age. Histopathology reveals expansion of thymic epithelial and lymphoid compartments (right) as observed routinely in *TgK18GT_121_; β-actin Cre* mice.
(TIF)

Figure S12 **Thymic phenotype in** ***TgK18GT_121_*** **;** ***β-actin Cre*** **mice.** A. Kaplan-Meier survival curve analysis of both *TgK18GT_121_* lines crossed to *β-actin Cre* mice. B. T_121_ (green) was highly induced and coexpressed with K18 (red) in thymus by IF shown as confocal merged image. C. Proliferation and apoptosis levels were assessed by Ki-67 and TUNEL, respectively, in wildtype (WT) and *TgK18GT_121_; β-actin Cre* thymus.
(TIF)

Figure S13 **Apoptosis in T_121_-expressing** ***TgK18GT_121_;*** ** ***β-actin Cre*** **tissues.** Apoptosis, evaluated as in Figure S9, was low in most tissues of 2 months mice. Scale bar = 50 µM.
(TIF)

Figure S14 **T_121_ induction via tamoxifen injection in** ***TgK19GT_121_;K19CreER*** **mice.** A. T_121_ (brown) was readily detected in 4–6 weeks post induced tissues. Scale bar = 50 µM. B. T_121_ (green) was co-expressed with endogenous K19 (red) by IF and shown as confocal merged images. Scale bar = 25 µM.
(TIF)

Table S1 Summary of endogenous K18 and K19 expression in wildtype mice, eGFP expression in *TgK18GT_121_* and *TgK19GT_121_* mice, and T_121_ expression in *TgK18GT_121_*; *β-actin Cre* ^1^, *TgK19GT_121_*; *β-actin Cre* ^2^, and *TgK19GT_121_*;*K19CreER* ^3^ by immunostaining.
(DOC)

Table S2 Summary of live born pups from both lines of *TgK19GT_121_* heterozygous crossed to *β-actin Cre* homozygous mice.
(DOC)
