# Supplementary figures and images for: Carcinoma Initiation via Rb Tumor Suppressor Inactivation: A Versatile Approach to Epithelial Subtype-Dependent Cancer Initiation in Diverse Tissues
Source: PLoS One. 2013 Dec 2;8(12):e80459. doi: 10.1371/journal.pone.0080459 (PMC3846618; doi:10.1371/journal.pone.0080459)

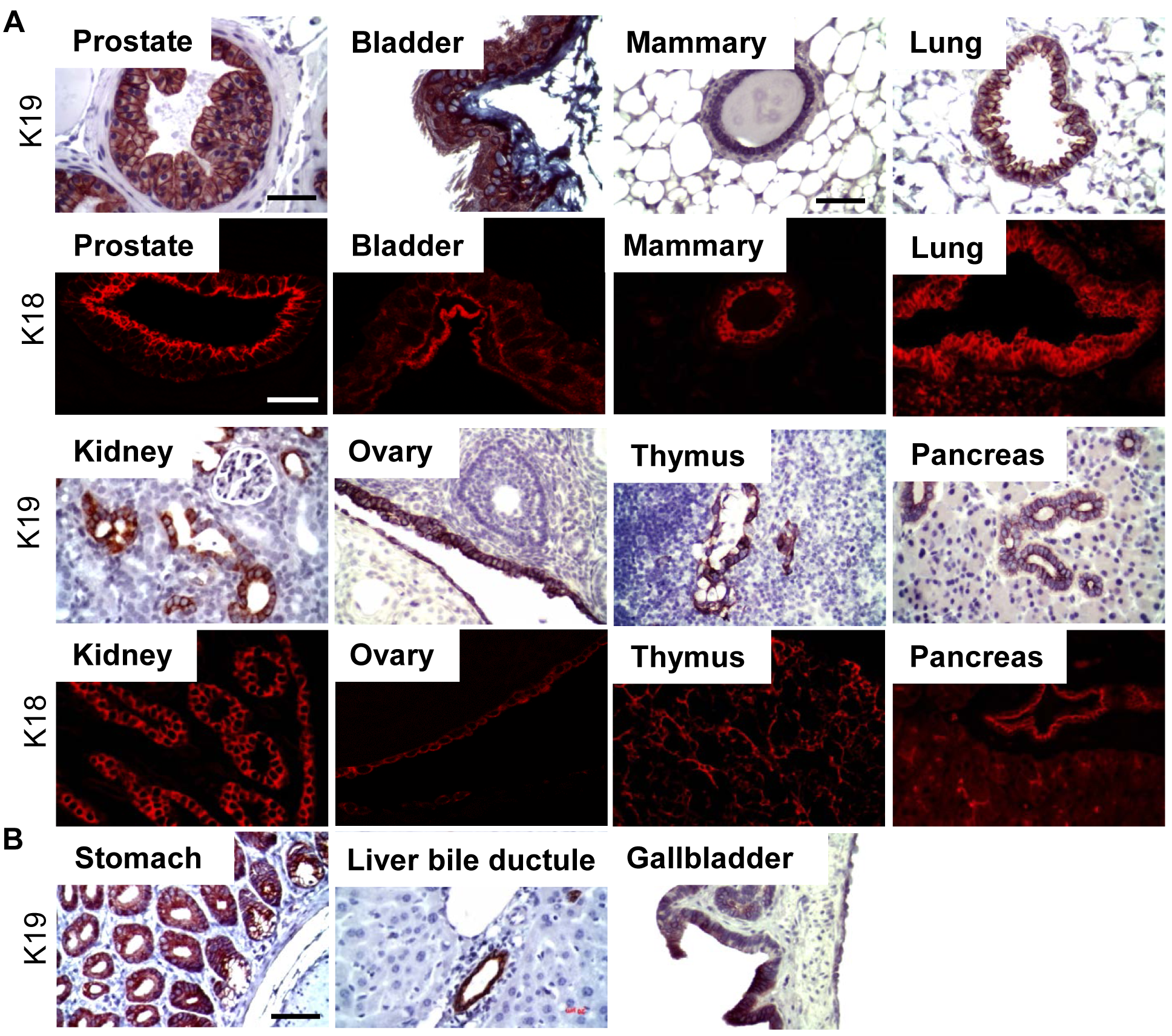

Supplement: Figure S1 — Endogenous K19 and K18 expression in wildtype mice. A. Endogenous K19 (brown, Scale bar = 50 µM) and K18 (red, Scale bar = 25 µM) expression was detected by immunohistochemistry (IHC) and immunofluorescence (IF), respectively. B. Endogenous K19 expression (brown) was detected by IHC. Scale bar = 50 µM. (TIF) [file pone.0080459.s001.tif]

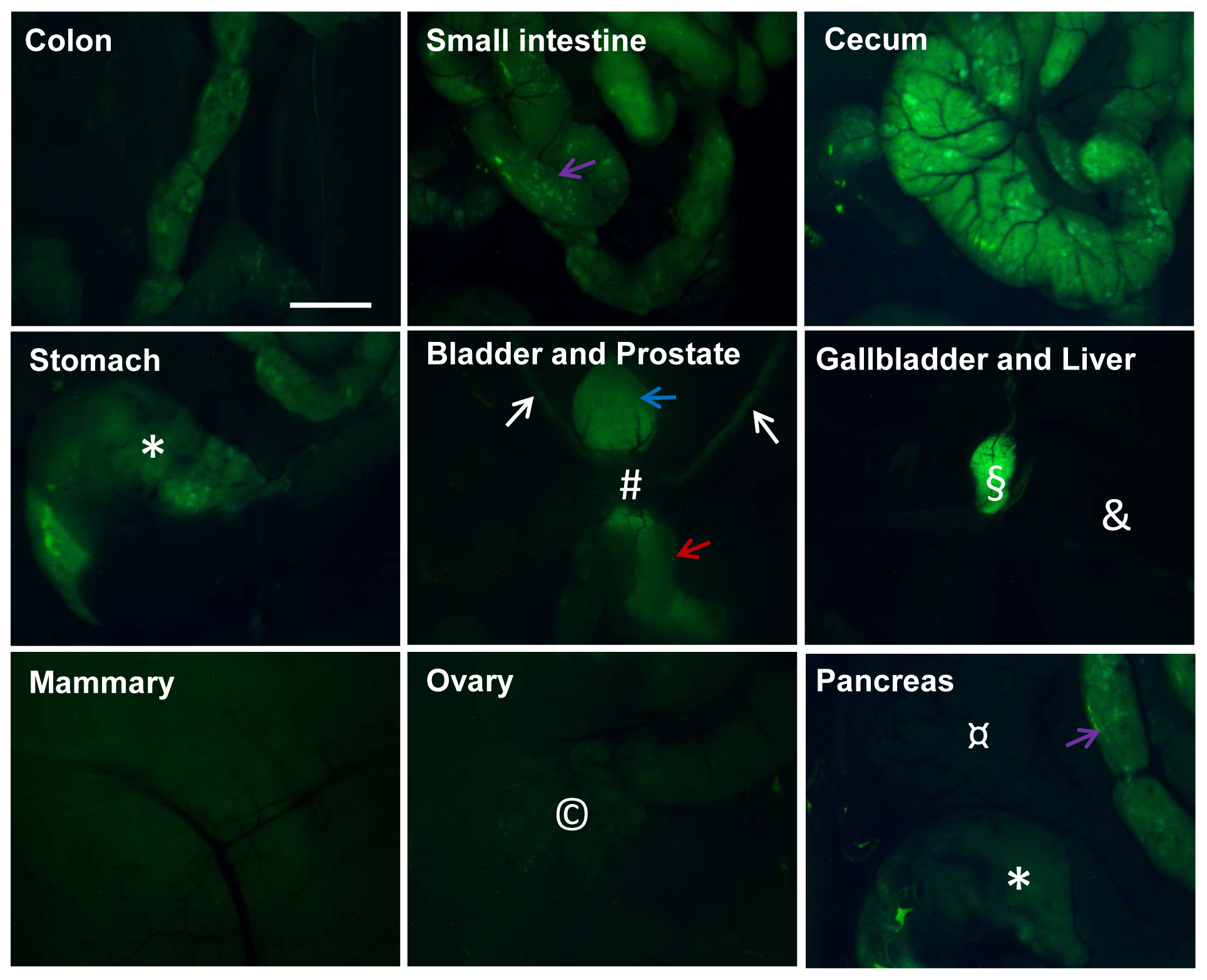

Supplement: Figure S2 — GFP expression in TgK19GT121 tissues. GFP fluorescence was visualized using SteREO Discovery.V20 Zeiss microscope. Indicated regions with grossly detectable expression include colon, cecum, small intestine (purple arrow), stomach (*), urinary bladder (blue arrow), vas deferens (white arrows), seminal vesicle (red arrow), and gallbladder (§). Expression was not observed by this method in prostate (#), liver (&), mammary gland, ovary (©), and pancreas (¤). Scale bar = 2000 µM. (TIF) [file pone.0080459.s002.tif]

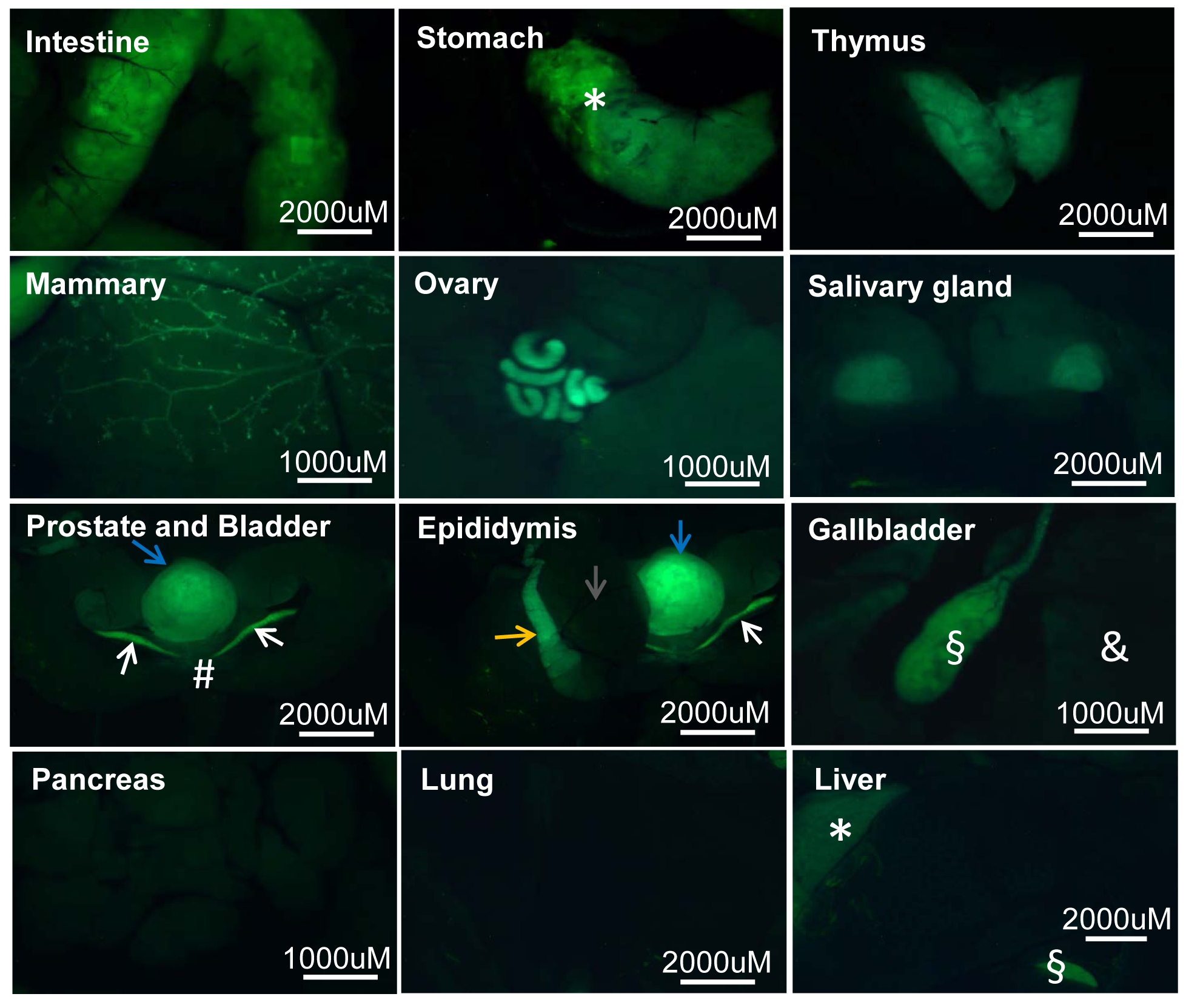

Supplement: Figure S3 — GFP expression in TgK18GT121 tissues. GFP fluorescence was observed in intestine (small intestine, cecum, and colon), stomach (*), thymus, mammary gland, ovary, salivary gland, urinary bladder (blue arrows), vas deferens (white arrows), epididymis (yellow arrow), gallbladder (§), but not in prostate (#), testis (gray arrow), pancreas, lung, and liver (&) using SteREO Discovery.V20 Zeiss microscope. (TIF) [file pone.0080459.s003.tif]

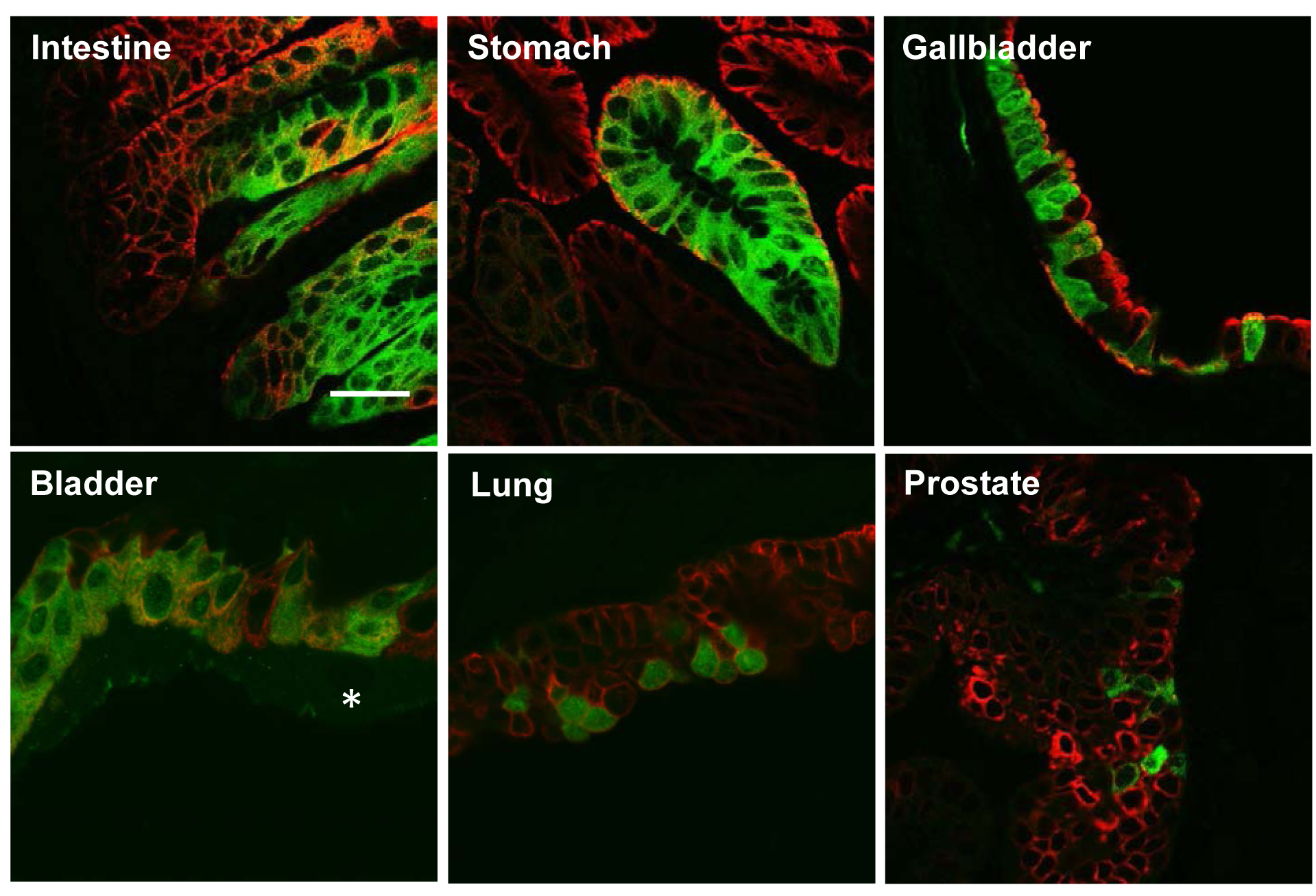

Supplement: Figure S4 — Co-expression of transgenic GFP and endogenous K19 in TgK19GT121 tissues. GFP (green) and endogenous K19 (red) were co-stained by IF and shown as confocal merged images. Umbrella cells (*) in urinary bladder epithelium were negative for K19 and GFP. Scale bar = 25 µM. (TIF) [file pone.0080459.s004.tif]

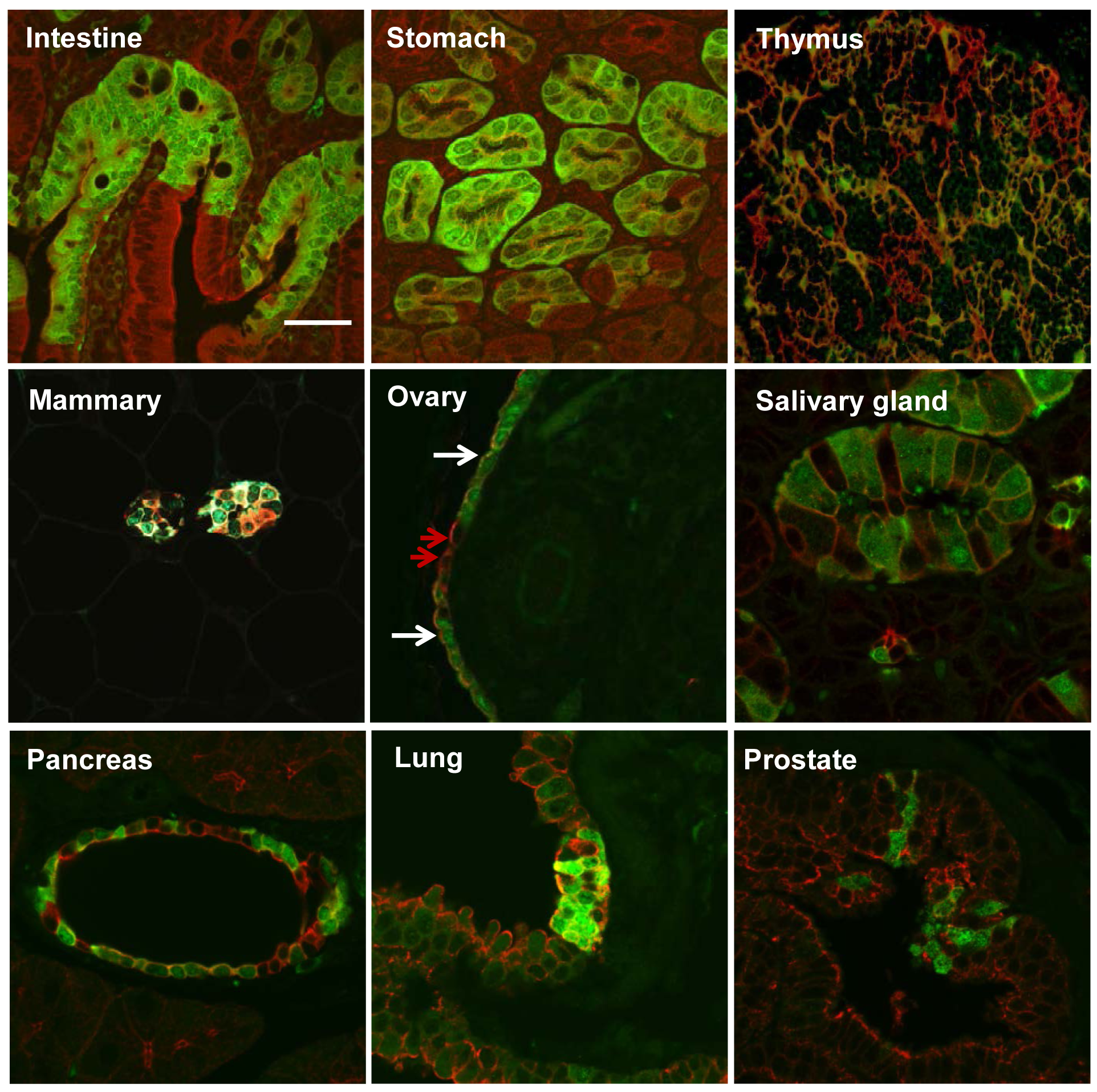

Supplement: Figure S5 — Co-expression of transgenic GFP and endogenous K18 in TgK18GT121 tissues. GFP (green) and endogenous K18 (red) were co-stained by IF and shown as confocal merged images. White arrows indicate ovarian surface epithelial cells (OSECs), which co-expressed K18 and eGFP. Red arrows indicate OSECs that are negative for transgenic eGFP, but positive for endogenous K18, indicating mosaic expression typical of transgenes and observed to varying extents in all tissues. Scale bar = 25 µM. (TIF) [file pone.0080459.s005.tif]

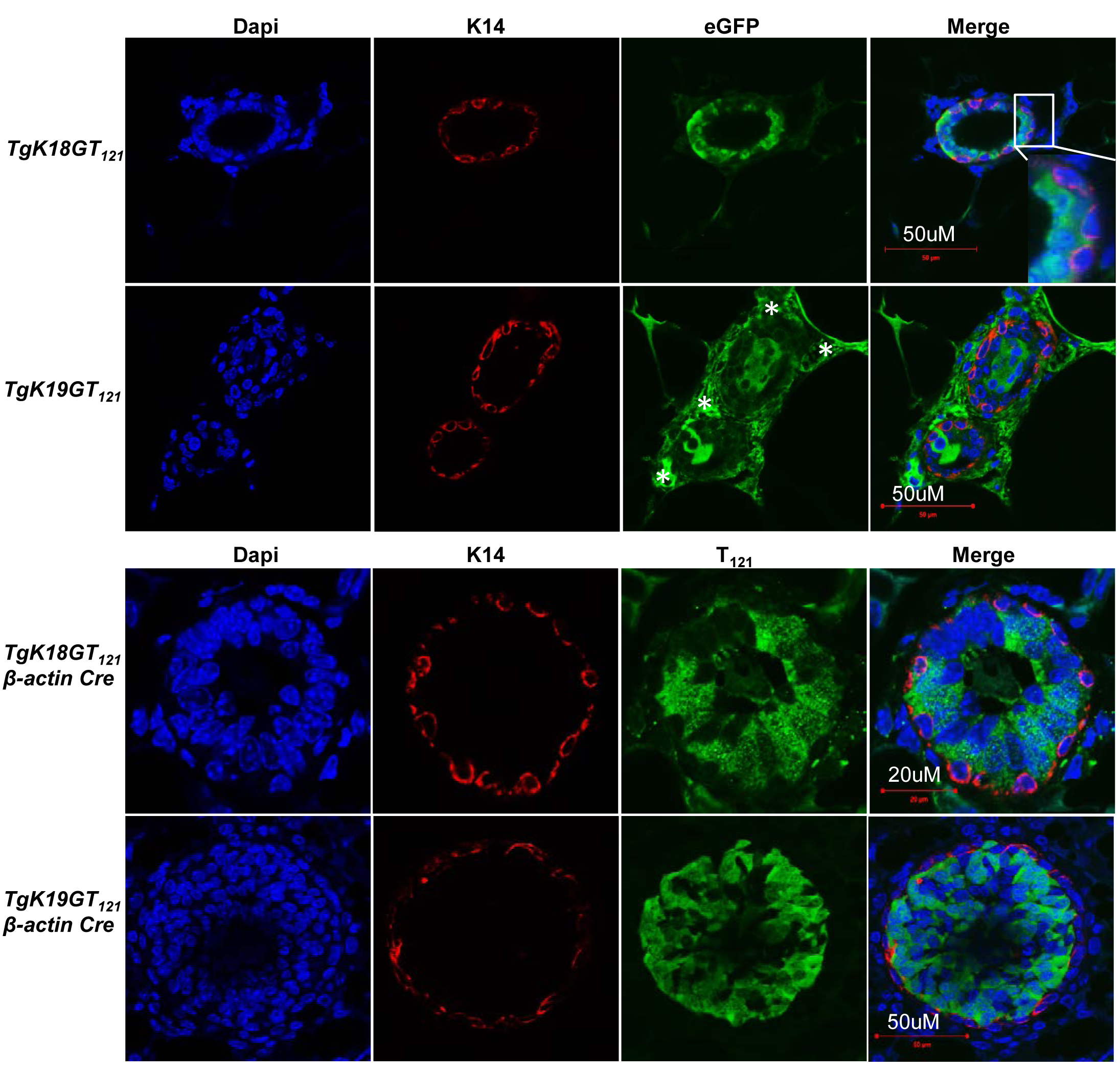

Supplement: Figure S6 — Exclusion of transgene expression from K14 cells in mammary glands. K14 was double immunostained with eGFP in TgK18GT121 and TgK19GT121 or T121 in TgK18GT121; β-actin Cre and TgK19GT121; β-actin Cre mammary glands. Dapi was used to counterstain nuclei. *indicates the high background staining of eGFP in mammary glands of TgK19GT121 mice. (TIF) [file pone.0080459.s006.tif]

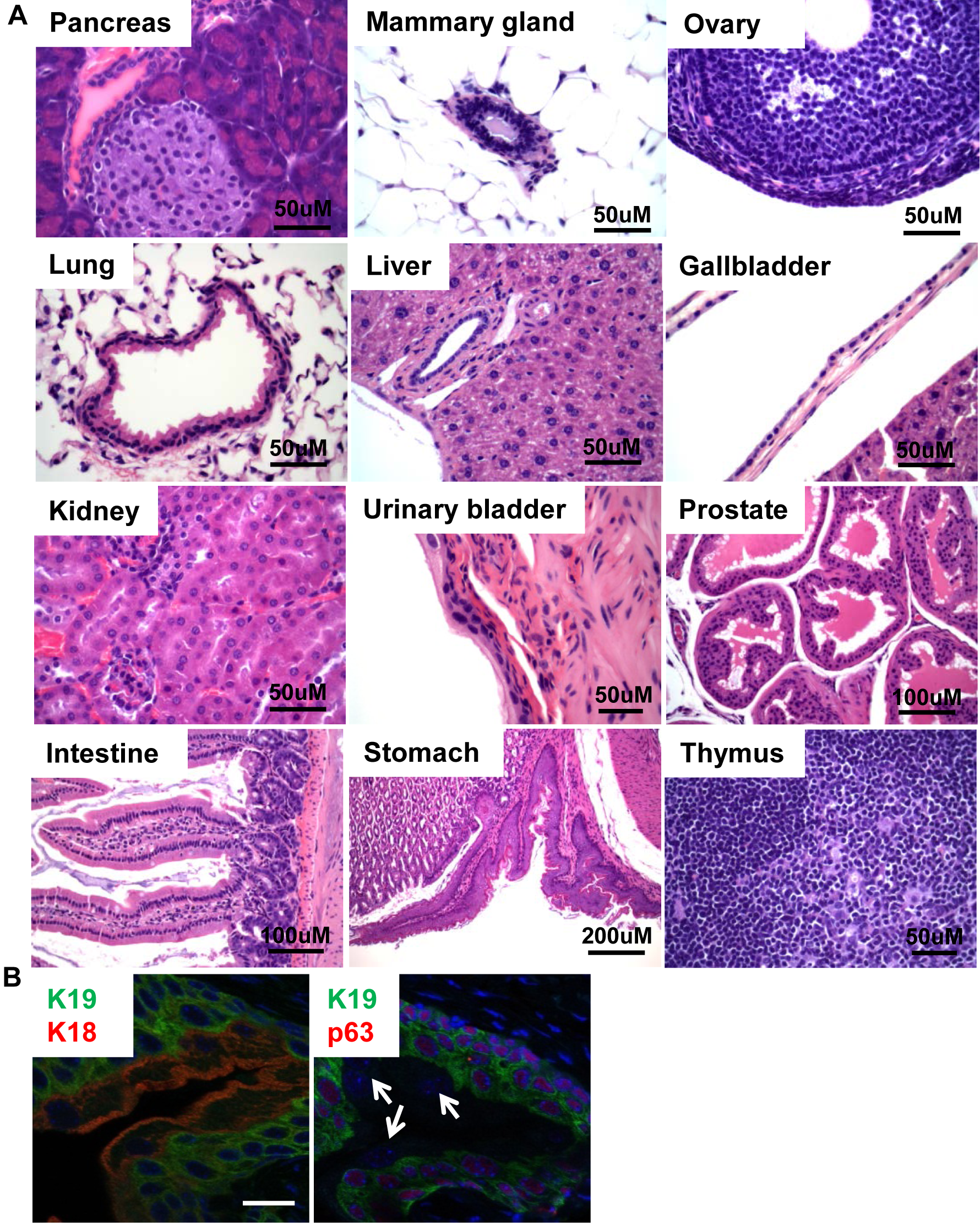

Supplement: Figure S7 — Wildtype mouse tissue architecture. A. Histology of wildtype tissues by H&E staining. B. Double immunostaining of K19 and K18 or K19 and p63 in urinary bladder. White arrows indicate umbrella cells negative for both K19 and p63. Scale bar = 25 µM. (TIF) [file pone.0080459.s007.tif]

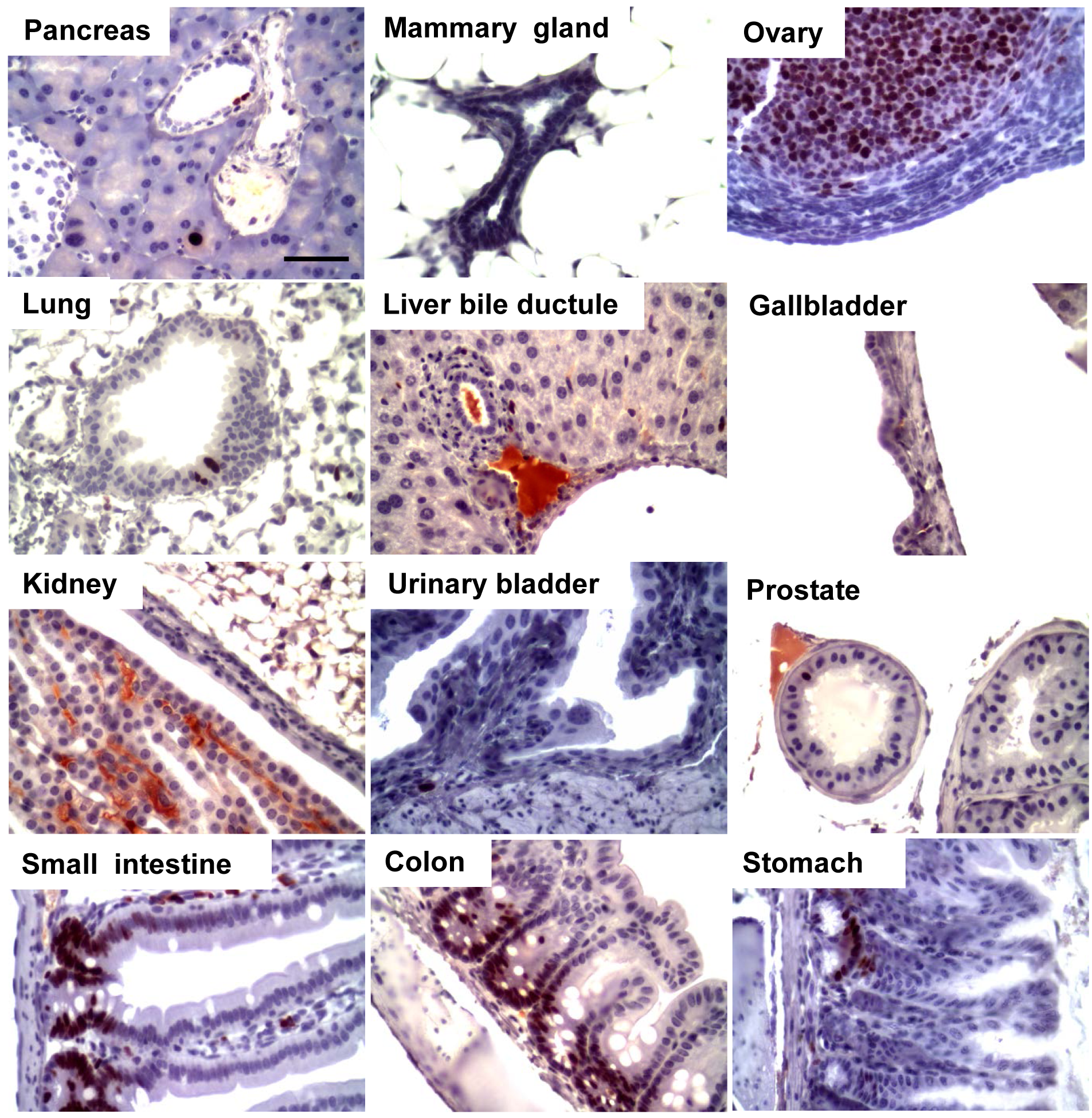

Supplement: Figure S8 — Proliferation in wildtype tissues. Proliferation was assessed by Ki-67 IHC (brown) and was either low or undetectable in most tissues. Normal small intestine, colon, stomach, and ovarian follicle proliferation was readily detected. Scale bar = 50 µM. (TIF) [file pone.0080459.s008.tif]

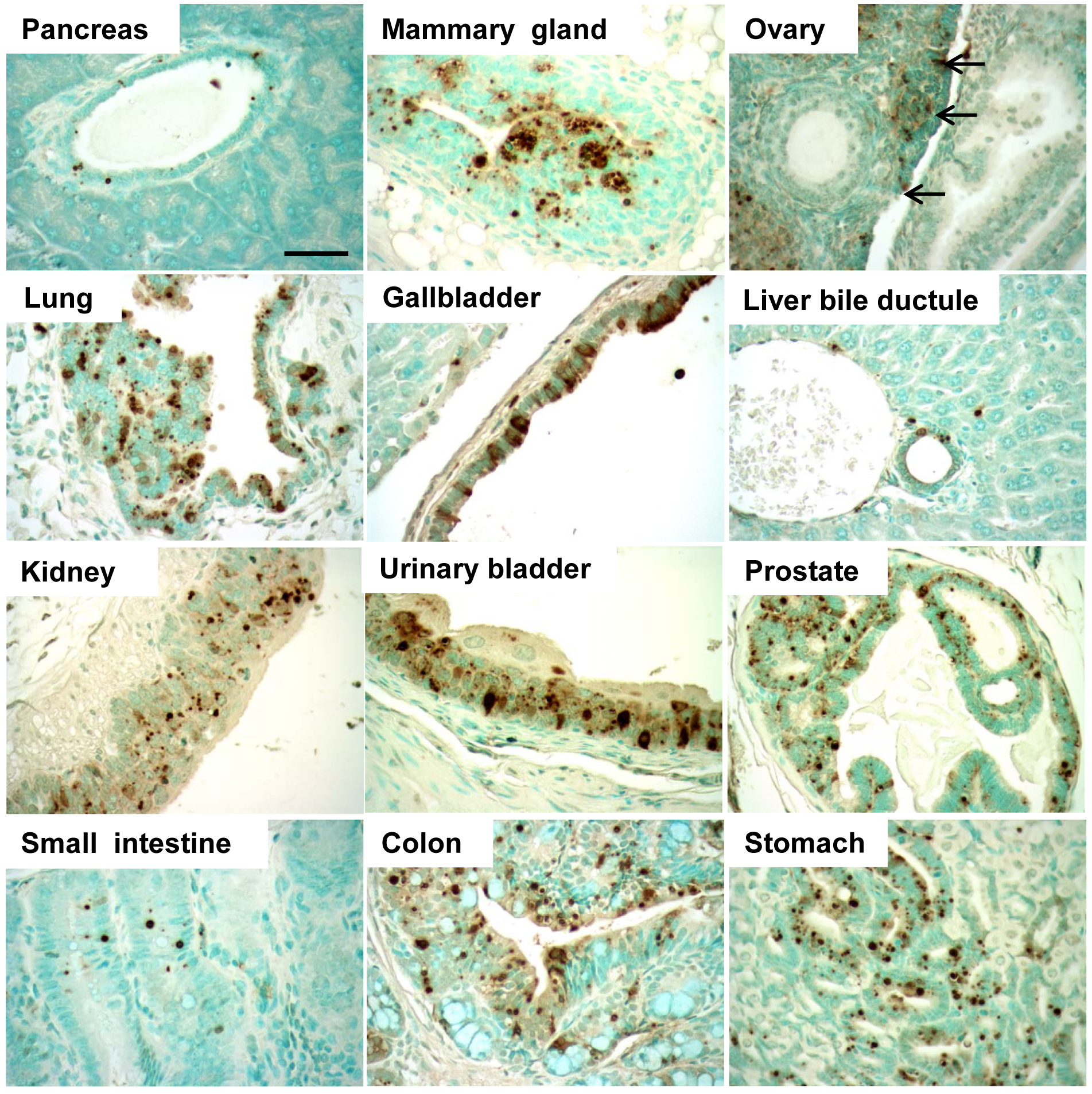

Supplement: Figure S9 — Apoptosis in T121-expressing TgK19GT121; β-actin Cre tissues. Apoptosis (brown) was assessed by TUNEL in tissues of 2 months old mice. Methyl green was used to counterstain nuclei. Scale bar = 50 µM. (TIF) [file pone.0080459.s009.tif]

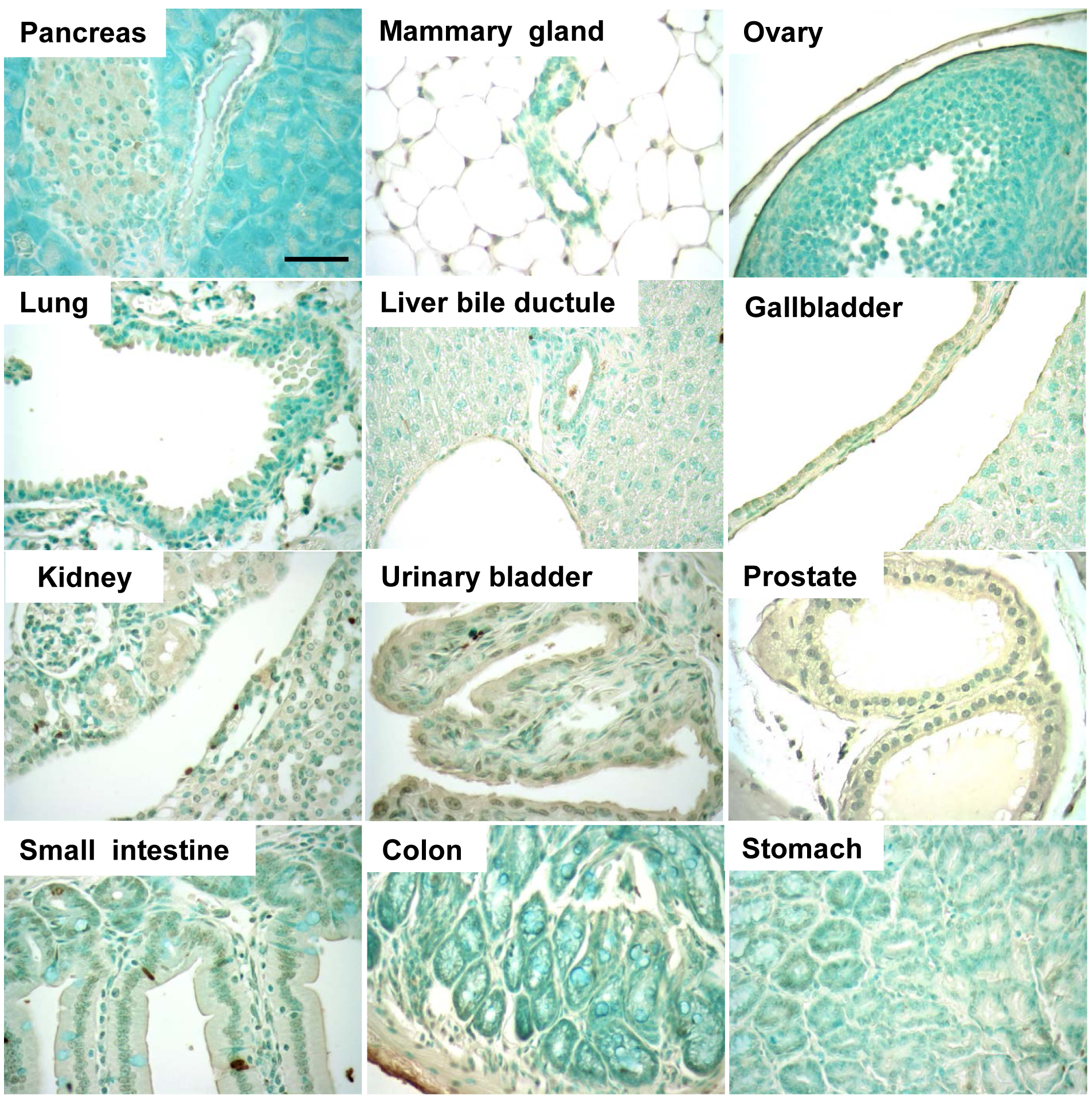

Supplement: Figure S10 — Apoptosis in wildtype tissues. Apoptosis, evaluated as in Figure S9, was minimal in tissues of 2 months old wildtype mice. Scale bar = 50 µM. (TIF) [file pone.0080459.s010.tif]

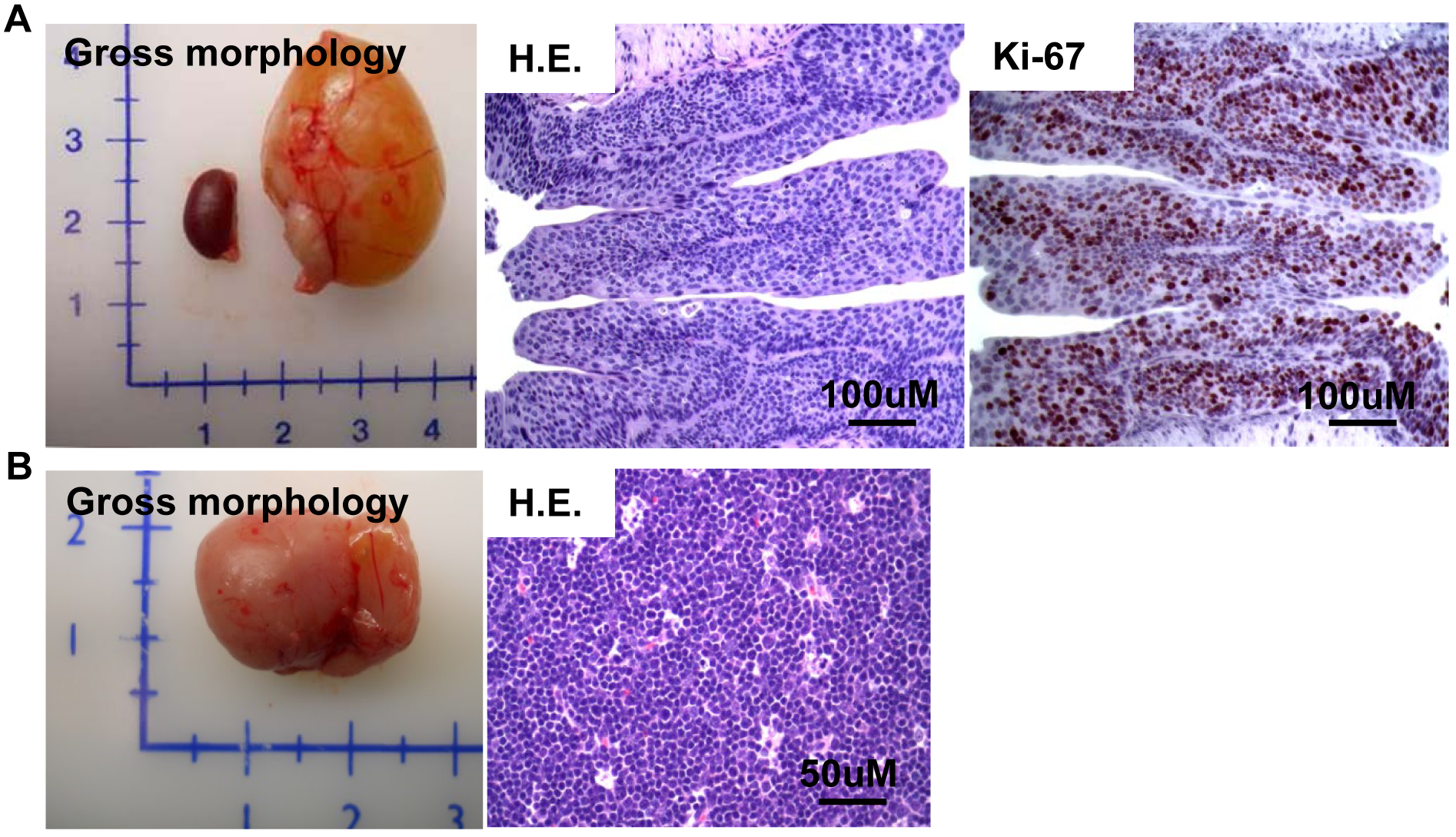

Supplement: Figure S11 — Renal and thymic phenotypes in TgK19GT121; β-actin Cre mice. A. Left: Gross morphology of unilateral hydronephrosis shows relative normal size of left kidney and cystic right kidney; Middle: Histopathology of the renal pelvis/ureter junction revealed by H&E staining; Right: proliferation of the renal pelvis/ureter junction by Ki-67 IHC. Marked hyperplasia of transitional cell epithelium in the junction resulted in obstruction of urine outflow from the kidney. Most mice were euthanized due to hydronephrosis at 2–7 months. B. Some TgK19GT121-34; β-actin Cre mice also developed life-threatening thymic masses (left) at 7 months of age. Histopathology reveals expansion of thymic epithelial and lymphoid compartments (right) as observed routinely in TgK18GT121; β-actin Cre mice. (TIF) [file pone.0080459.s011.tif]

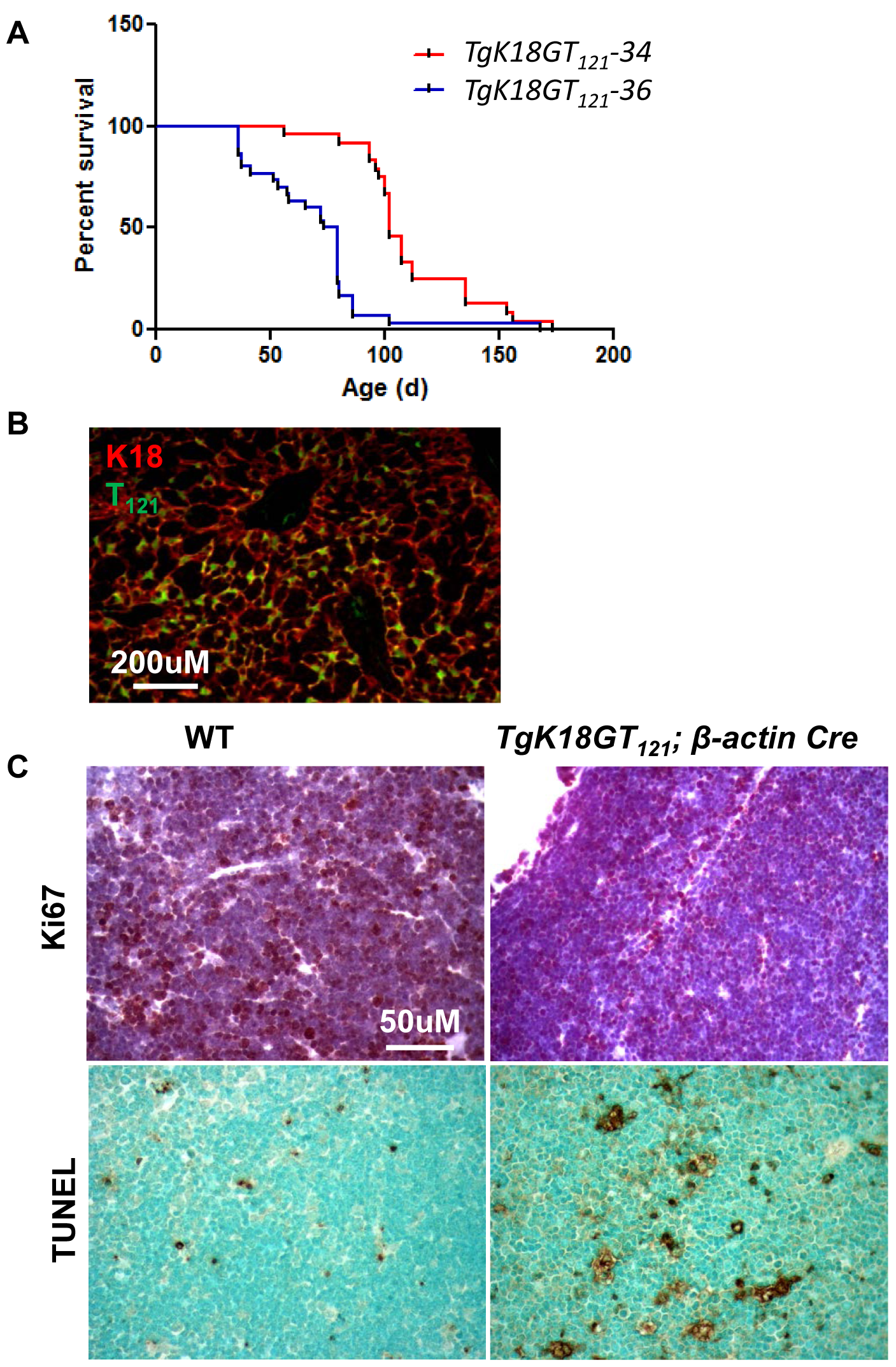

Supplement: Figure S12 — Thymic phenotype in TgK18GT121 ; β-actin Cre mice. A. Kaplan-Meier survival curve analysis of both TgK18GT121 lines crossed to β-actin Cre mice. B. T121 (green) was highly induced and coexpressed with K18 (red) in thymus by IF shown as confocal merged image. C. Proliferation and apoptosis levels were assessed by Ki-67 and TUNEL, respectively, in wildtype (WT) and TgK18GT121; β-actin Cre thymus. (TIF) [file pone.0080459.s012.tif]

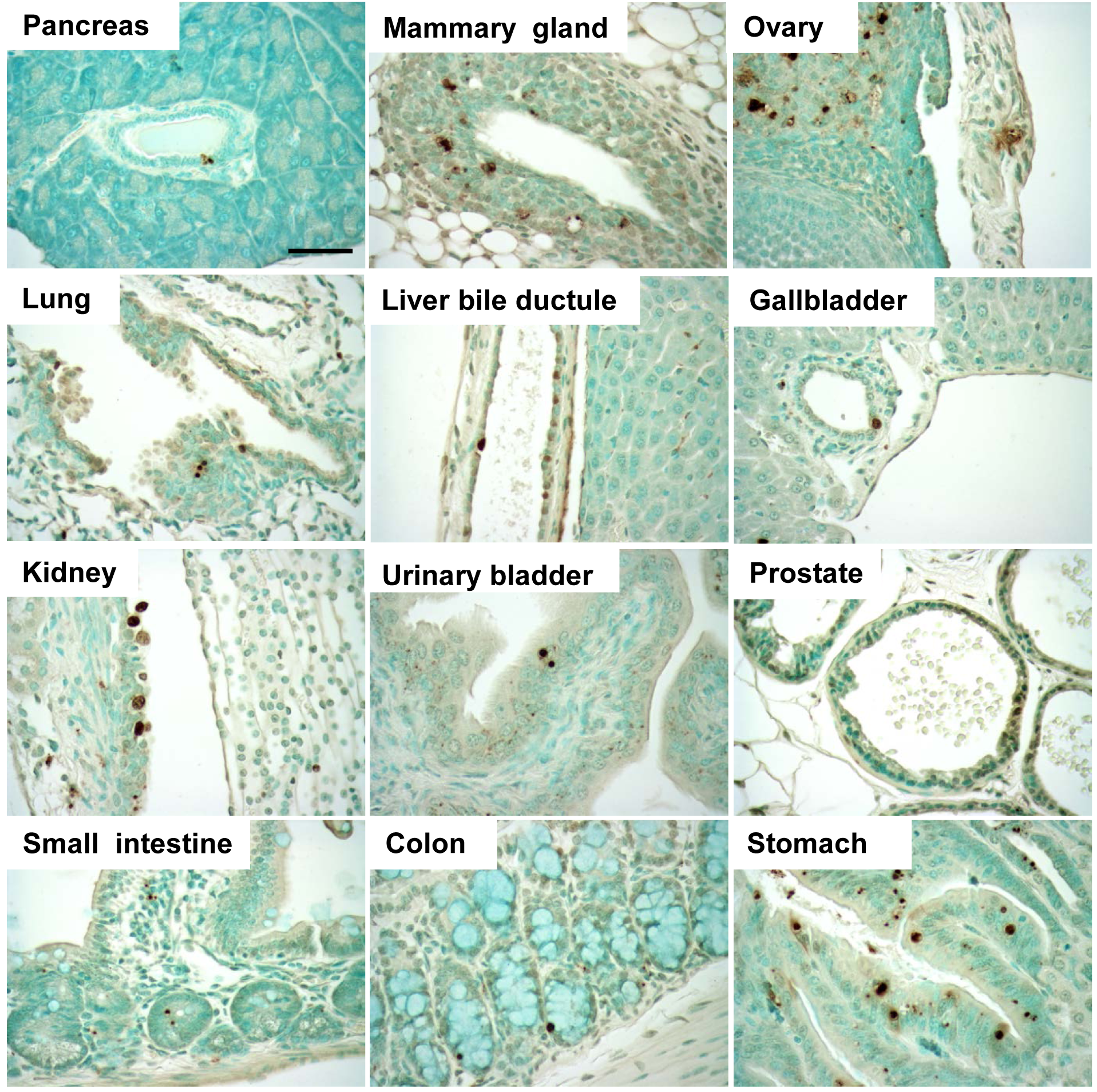

Supplement: Figure S13 — Apoptosis in T121-expressing TgK18GT121; β-actin Cre tissues. Apoptosis, evaluated as in Figure S9, was low in most tissues of 2 months mice. Scale bar = 50 µM. (TIF) [file pone.0080459.s013.tif]

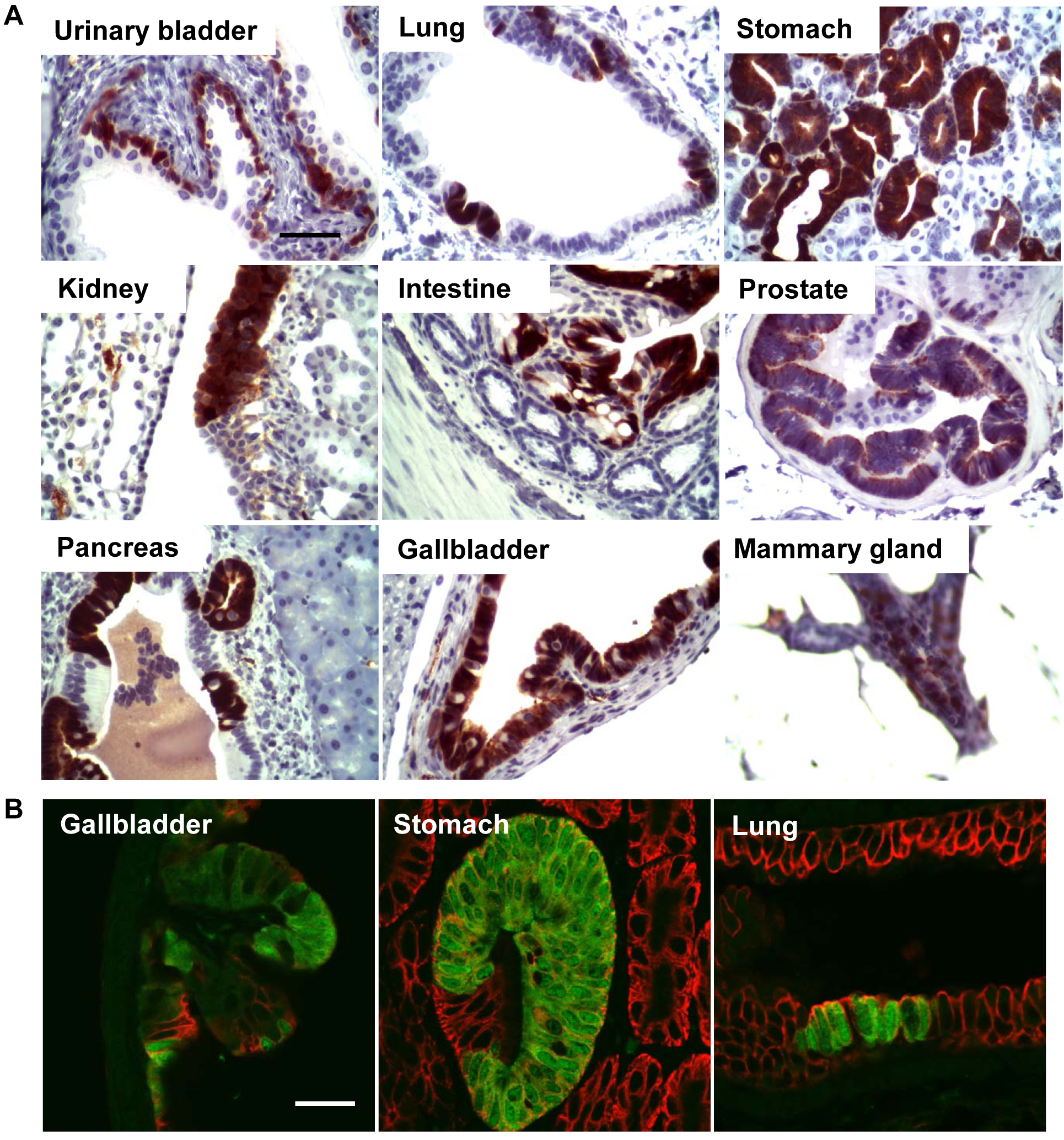

Supplement: Figure S14 — T121 induction via tamoxifen injection in TgK19GT121;K19CreER mice. A. T121 (brown) was readily detected in 4–6 weeks post induced tissues. Scale bar = 50 µM. B. T121 (green) was co-expressed with endogenous K19 (red) by IF and shown as confocal merged images. Scale bar = 25 µM. (TIF) [file pone.0080459.s014.tif]
