# Supplementary material for: Carcinoma Initiation via Rb Tumor Suppressor Inactivation: A Versatile Approach to Epithelial Subtype-Dependent Cancer Initiation in Diverse Tissues
Source: PLoS One. 2013 Dec 2;8(12):e80459. doi: 10.1371/journal.pone.0080459 (PMC3846618; doi:10.1371/journal.pone.0080459)
Supplement: Table S1 — Summary of endogenous K18 and K19 expression in wildtype mice, eGFP expression in TgK18GT121 and TgK19GT121 mice, and T121 expression in TgK18GT121; β-actin Cre 1, TgK19GT121; β-actin Cre 2, and TgK19GT121;K19CreER 3 by immunostaining. (DOC) [file pone.0080459.s015.doc]

**Table S1. Summary of endogenous K18 and K19 expression in wildtype mice, eGFP expression in *TgK18GT_121_* and *TgK19GT_121_* mice, and T_121_ expression in *TgK18GT_121_*;*β-actin Cre*^1^, *TgK19GT_121_*;*β-actin Cre*^2^, and *TgK19GT_121_*;*K19CreER*^3^ by immunostaining.**

| **Tissues** | **Endogenous K18** | **Endogenous K19** | **eGFP in *TgK18GT_121_*** | **T_121_^1^** | **eGFP in *TgK19GT_121_*** | **T_121_^2^** | **T_121_^3^** |
| --- | --- | --- | --- | --- | --- | --- | --- |
| **Prostate** | + (Lu) | + (Lu) | + | + | + | +++ | +++ |
| **Mammary gland** | + (Lu) | + (Lu) | +++ | +++ | + | +++ | ++ |
| **Ovary** | + (OSECs) | + (OSECs) | +++ | ++ | - | +++ | + |
| **Renal pelvis** | + | + | +++ | + | ++ | +++ | +++ |
| **Liver bile ductules** | + | + | +++ | - | + | +++ | + |
| **Lung, bronchioles** | + | + | + | - | + | +++ | ++ |
| **Gallbladder** | + | + | +++ | - | ++ | +++ | +++ |
| **Pancreas, ducts** | + | + | +++ | + | - | +++ | ++ |
| **Intestine** | + | + | ++ | ++ | +++ | +++ | +++ |
| **Stomach, glandular** | + | + | +++ | ++ | +++ | +++ | +++ |
| **Salivary gland, ducts** | + | + | +++ | - | - | + | - |
| **Thymic epithelial cells** | + | + | +++ | +++ | - | + | - |
| **Urinary bladder** | +* | +** | +++ | + | +++ | +++ | ++ |

Endogenous K18 and K19 expression were scored as + if positive staining was observed, and – if there was no positive staining. eGFP and T_121_ expression were scored as + (low) to +++ (high), or – (no expression or under current detection level). Lu: luminal cells. OSECs: ovarian surface epithelial cells. *Umbrella cells; **Intermediate and basal cells.
