# Supplementary material for: Carcinoma Initiation via Rb Tumor Suppressor Inactivation: A Versatile Approach to Epithelial Subtype-Dependent Cancer Initiation in Diverse Tissues
Source: PLoS One. 2013 Dec 2;8(12):e80459. doi: 10.1371/journal.pone.0080459 (PMC3846618; doi:10.1371/journal.pone.0080459)
Supplement: Table S2 — Summary of live born pups from both lines of TgK19GT121 heterozygous crossed to β-actin Cre homozygous mice. (DOC) [file pone.0080459.s016.doc]

**Table S2. Summary of live born pups from both lines of *TgK19GT_121_* heterozygous crossed to *β-actin Cre* homozygous mice.**

| **Genotype** | **N (Female)** | **N (Male)** | **Total N (observed frequency)** | **Expected frequency** |
| --- | --- | --- | --- | --- |
| **TgK19GT_121_34-; Cre+** | 41 | 45 | 86 (66%) | 50% |
| **TgK19GT_121_34+; Cre+** | 20 | 24 | 44 (34%) | 50% |
| **TgK19GT_121_43-; Cre+** | 27 | 27 | 54 (55%) | 50% |
| **TgK19GT_121_43+; Cre+** | 24 | 20 | 44 (45%) | 50% |
